# Genetic predisposition to B-cell acute lymphoblastic leukemia at 14q11.2 is mediated by a *CEBPE* promoter polymorphism

**DOI:** 10.1038/s41375-018-0184-z

**Published:** 2018-07-06

**Authors:** James B. Studd, Minjun Yang, Zhenhua Li, Jayaram Vijayakrishnan, Yi Lu, Allen Eng-Juh Yeoh, Kajsa Paulsson, Richard S. Houlston

**Affiliations:** 1 Division of Genetics and Epidemiology, The Institute of Cancer Research, London, SM2 5NG United Kingdom; 2 Department of Laboratory Medicine, Division of Clinical Genetics, Lund University, 221 85 Lund, Sweden; 3 Centre for Translational Research in Acute Leukaemia, Department of Paediatrics, Yong Loo Lin School of Medicine, National University of Singapore, Singapore, Singapore; 4 Viva-University Children’s Cancer Centre, Khoo Teck Puat-National University Children’s Medical Institute, National University Hospital, National University Health System, Singapore, Singapore; 5 Division of Molecular Pathology, The Institute of Cancer Research, London, SM2 5NG United Kingdom

**Keywords:** Cancer genetics, Acute lymphocytic leukaemia

## Abstract

Acute lymphoblastic leukaemia (ALL) is the most common paediatric malignancy. Genome-wide association studies have shown variation at 14q11.2 influences ALL risk. We sought to decipher causal variant(s) at 14q11.2 and the mechanism of tumorigenesis. We show rs2239630 G>A resides in the promoter of the CCAT enhancer-binding protein epsilon (*CEBPE*) gene. The rs2239630-A risk allele is associated with increased promotor activity and *CEBPE* expression. Depletion of *CEBPE* in ALL cells reduces cell growth, correspondingly CEBPE binds to the promoters of electron transport and energy generation genes. RNA-seq in *CEBPE* depleted cells demonstrates CEBPE regulates the expression of genes involved in B-cell development (*IL7R*), apoptosis (*BCL2*), and methotrexate resistance (*RASS4L*). *CEBPE* regulated genes significantly overlapped in *CEBPE* depleted cells, ALL blasts and IGH-CEBPE translocated ALL. This suggests *CEBPE* regulates a similar set of genes in each, consistent with a common biological mechanism of leukemogenesis for rs2239630 associated and *CEBPE* translocated ALL. Finally, we map IGH-CEBPE translocation breakpoints in two cases, implicating RAG recombinase activity in their formation.

## Introduction

Acute lymphoblastic leukemia (ALL) is the most common pediatric cancer in western countries. While the aetiology of ALL is poorly understood evidence implicates initiating transforming events occuring in utero [1–3], with secondary events required for transition to malignancy [4].

Our understanding of ALL susceptibility has been transformed by genome-wide association studies (GWAS) [5–8]. GWAS have identified 11 loci associated with ALL, including the 14q11.2 risk locus, which has been replicated in multiple independent series [9, 10]. ALL risk loci often map in the vicinity of B-cell development genes, including *IKZF1*, *GATA3*, *ARID5B* and at 14q11.2 mapping to CCAT enhancer-binding protein epsilon (*CEBPE*) [6], suggesting a central role for dysregulated B-cell development in leukemogenesis. Correspondingly, crucial B-cell development transcription factors *IKZF1*, *PAX5*, and *EBF1* are also the targets of frequent somatic mutation [11, 12], highlighting that GWAS signals and somatic mutations may impact upon the same genes.

Around 60% of BCP-ALL is characterised by translocations, most common of which, *t*(12:21) produces the chimeric, ETV6-RUNX1, transcription factor. Three percent of BCP-ALL features a translocation at the immunoglobulin heavy chain (IGH) locus that often involves a *CEBP* family member. These juxtapose IGH with the coding region of *CEBP*, increasing *CEBP* expression [13], raising the possibility that the 14q11.2 GWAS association is also mediated through increased *CEBPE* expression.

We sought to identify the causal polymorphism(s) for the 14q11.2 association. Our data are compatible with rs2239630 in the promoter of *CEBPE* driving the association and increasing *CEBPE* expression.

## Material and methods

### Ethics

Samples and clinicopathological information were collected with ethical board approval. ALL blast RNA sequencing and genotyping sample collection was approved by the Lund University, nos 2011/289 and 2017/796. Informed consent was granted from all participants.

### GWAS

UK GWAS and German GWAS have been previously reported [6, 7]. Briefly, the UK GWAS comprised 824 cases, genotyped using Illumina Human 317 K arrays; WTCC2 controls were of 2699 of 1958 British Birth Cohort and 2501 from the UK Blood Service. German GWAS comprised 834 cases, genotyped using Illumina Human OmniExpress-12v1.0 arrays. Controls comprised 2024 individuals from the Heinz Nixdorf Recall study [14]. GWAS QC has been described previously [7]. Untyped genotypes were imputed using IMPUTE2 v2.3 [15] with combined UK10K (ALSPAC and TwinsUK) [16] and 1000 Genomes Project (phase III) [17] references. Poorly imputed SNPs (INFO score <0.8) were excluded. Association between SNP and ALL was performed using SNPTESTv2.5 [18]. Meta-analysis was undertaken using META v1 [18].

### Hi-C data analysis

Hi-C data in Supplementary Fig.1 derived from GM12878 (MboI plus replicate) was obtained from (https://www.aidenlab.org/juicebox/) [19]. Data were viewed using balanced Knight-Ruiz normalisation at 5 kb resolution. Hi-C data in Supplementary Fig.7 (GM12878 and human embryonic stem cells) were from (http://yunliweb.its.unc.edu/HUGIn/; GSE87112).

### Cell lines and lentiviral transduction

REH, NALM6, and SEM B-ALL, Jurkat T-ALL cell lines were obtained from DMSZ (Braunschweig Germany). GM12815 and GM12760 were obtained from the Coriell Institute (Camden NJ, US). Cell lines were maintained at 37 °C, with 5% CO_2_ in RPMI with 10% FBS and GlutaMAX (Thermo Fisher Scientific). Doxycyclin inducible *CEBPE* shRNA knockdown, *CEBPE* and *ZNF148* overexpressing cell lines were generated by lentiviral transduction using *CEBPE* (V3THS_150517(A13), V3THS_404312(G3)), Empty, Non-targeting pTRIPZ or pCW57.1 vectors (GE Life Sciences or from David Root Addgene plasmid # 41393) into REH. Lentiviral particles were produced in HEK239T cells as described [20, 21]. Transduced REH cells were selected with 750 ng/ml puromycin for 1 week. Polyclonal populations were used for all assays. Cells were tested for mycoplasma using Promokine PCR Mycoplasma Test Kit I/C, no positive results were obtained. Cell identity was confirmed using Promega PowerPlex 16 microsatellite testing kit.

### Plasmid construction and luciferase assays

The *CEBPE* promoter containing rs2239630, rs2239632 and rs2239633 and *ZNF148* coding sequence cloned in pGL3 Promoter Vector (Promega) or pCW57.1 doxycycline inducible vectors, respectively. For luciferase assays, cells were electroporated and promoter activity was assayed using a Dual-Luciferase assay (Promega). Full methods are described in Supplementary material.

### Genotyping and sequencing

PCR for sequencing was carried out using Thermoprime DNA polymerase (Thermo Fisher Scientific). BigDye v3.1 sequencing reactions were analysed on an ABI7900HT (Applied Biosystems) (Primers in Supplementary Table 1). The concordance between imputation of rs2239630 and 133 sequenced genotypes was 96% (*R*^2^ = 0.90).

### ChIP-seq data mining, Blueprint, and Roadmap chromatin state dynamics

B-cell epigenetic profiles were obtained from Roadmap [22] and Blueprint [23] projects and ENCODE. Chromatin state dynamics (ChomHMM) for E031 is from (http://egg2.wustl.edu/roadmap/data/byFileType/chromhmm Segmentations/ChmmModels/coreMarks/jointModel/final/) and for GM12878 from (http://hgdownload.cse.ucsc.edu/goldenPath/hg19/encodeDCC/wgEncodeBroadHmm/ wgEncodeBroadHmmGm12878HMM.bed.gz). DNAse hypersensitivity data from the ALL blast pz294 (European Genome-phenome Archive EGAD00001002499) is from (ftp://ftp.ebi.ac.uk/pub/databases/blueprint/data/homo_sapiens/GRCh38/bone_marrow/pz_294/Acute_Lymphocytic_Leukemia_CTR/DNase-Hypersensitivity/NCMLS/) and for GM12878 from (http://hgdownload.cse.ucsc.edu/goldenPath/hg19/encodeDCC/wgEncodeUwDnase/ wgEncodeUwDnaseGm12878HotspotsRep2.broadPeak.gz). Proteins binding SNP rs2239630 extracted from Encode (http://hgdownload.cse.ucsc.edu/goldenPath/hg19/encodeDCC/wgEncodeRegTfbsClustered/wgEncodeRegTfbsClusteredV3) and ChIP Atlas (http://chip-atlas.org) ChIP-seq data. REH H3K27ac ChIP-Seq data were obtained from GSE84052 (https://www.ncbi.nlm.nih.gov/geo/query/acc.cgi?acc = GSE84052).

### ChIP

Cells were fixed in 1% formaldehyde in media for 10 min at RT with agitation and quenched in 125 mM glycine. ChIP was performed using the Active Motif ChIP-IT Express kit (Active Motif), except after mircococcal nuclease DNA digestion, cells were sonicated in a Biorupter (Diagenode) for 10 min on high 30 s ON/OFF at 4 °C. A total of 25 µg of chromatin was incubated with 2 µg of either anti-YY1 (Abcam ab12132), ELF1 (Santa Cruz sc-133096), MAX (Abcam ab53570), E2A (Santa Cruz sc133075), ZNF148 (Atlas Antibodies HPA001656), 0.5 µg of SPI1 (Life Technologies A13971) or an equal amount of control antibody, mouse IgG2b (Thermo Fisher Scientific MA5-14447) or rabbit IgG (AbCam ab37415) and incubated at 4 °C O/N with rotation. Target quantities were interpolated and normalised to input DNA (primers in Supplementary Table 2). rs2239630 allele-specific q-RT PCR primers demonstrated a 300-fold allele preference.

### ChIP-sequencing

ChIP-Seq sample preparation was performed as above, using 2 µg anti-CEBPE (Atlas Antibodies HPA002928), except: (1) After final ChIP wash, DNA was eluted in 100 µl of 1% SDS and 100 mM NaHCO_3_ for 15 min at RT with rotation. (2) After the addition of 4 µl of 5 M NaCl and protinase K digestion as per guidelines, duplicate reactions were pooled and purified using QIAquick PCR Purification columns (Qiagen). A total of 10 ng of ChIP’d and input DNA was used as material for Illumina NGS preparation using NEBNext ChIP-Seq Library preparation kit (New England Biolabs) and sequenced on a MiSeq using 150 bp Kit v3 (Illumina). Raw FASTQ ChIP-seq reads (CEBPE and H3K27ac) were mapped to human reference genome (human_g1k_v37) using Stampy v1.0.28, for H3K27ac data common (>1% frequency) SNPs (dbSNP build 150) were edited to ‘N’. BAM files were filtered to remove sequences with <MAPQ30 score. Peaks were called using MACS2 2.1.1 [24] against input DNA. Putative CEBPE-regulated genes are listed in Supplementary Table 7 (defined as those with an Ensembl 90 TSS within 1 kb of a peak). CEBPE ChIP motif discovery was performed using HOMER 4.9.1 [25].

### siRNA transfection and reverse transcription

A total of 4 × 10^6^ REH were electroporated with 200 nM of siRNA (Eurofins Genomics) (sequences in Supplementary Table 3) as described above and incubated for 24 h before lysis. RNA was prepared using the Qiagen RNAeasy kit. A total of 1–2 µg of RNA was reverse transcribed using MMLV (Promega) and 5 µM dT_15_.

### qRT-PCR

qRT-PCR was performed using SYBR Green (Thermo Fisher Scientific). Samples were analysed undiluted for ChIP or 1/100 for cDNA. Biological replicates contained three technical replicates. PCR-quantitation, by standard curve method. Fivefold serial dilutions of DNA or cDNA were used to control for differential primer efficiency. For siRNA assays target gene expression was normalised to the geometric average of *PPIA*, *TBP* or *PPIA*, *TUB*β as indicated.

### Electrophoretic mobility shift assay

EMSAs were performed as described in ref. [26] using rs2239630 allele-specific probes (5′-AGGCTGGTGCTTCGCCCCTC[A/G]CCCTGGGCCTGAGGCTCTGC-3′). Supershift assays were performed using anti-ZNF148 (Atlas Antibodies HPA001656) or IgG isotype control (AbCam ab37415). Full methods are described in Supplementary materials.

### Cell growth assay

Cell viability was quantified using a final concentration of 0.5 mg/ml MTT (3-(4,5-Dimethylthiazol-2-yl)-2,5-diphenyltetrazolium bromide, Sigma) at 37 °C for 2 h and reactions stopped with an equal volume of 10% SDS with 0.01% of 37% HCl. Plates were incubated for 24 h in the dark and absorbance read at 562 nm.

### Flow cytometry

For cell cycle analysis, around 10^6^ cells were suspended in 500 µl of −20 °C 70% v/v ethanol and fixed at −20 °C O/N, before resuspension in 500 µl ml of 1× propidium iodide (20 μg/ml (Sigma), 50 μg/ml RNase (Sigma) in PBS) for 2 h at RT. Data analysis was performed in FlowJo software (Tree Star Inc.) using the Watson Pragmatic algorithm. For annexin-V apoptosis assays ~100,000 cells were stained in 100 µl of buffer (10 mM HEPES, 140 mM NaCl, 2.5 mM CaCl_2_) with 5 µl of APC annexin-V (BD Pharmingen 550474) and 5 µl 20 µg/ml DAPI (4′,6-diamidino-2-phenylindole). Cells were stained for 15 min in the dark before the addition of 400 µl of buffer, all analyses were on a LSRII cytometer (Becton Dickinson).

### Cell line RNA-sequencing

shRNA expression was induced in REH cells with 1 μg/ml doxycycline (Sigma). Cells were lysed after 144 h and RNA prepared using Qiagen RNAeasy. *CEBPE* knockdown was verified by qRT-PCR. RNA integrity was assessed using a Tape Station 2200 (Agilent). Three biological replicates were processed each with two controls, (empty, non-targeting shRNA) and two *CEBPE* shRNAs (A13 and G3). Libraries were prepared using NEBNext Ultra II Directional RNA Library Prep Kit and sequenced on an Illuimna HiSeq 2500 using 2 × 101 version 4 paired end chemistry. RNA QC metrics are provided in Supplementary Table 4. FASTQs were filtered to remove adapter contamination using Trimmomatic v0.32 [27] and aligned to the human reference genome (human_g1k_v37) and transcript reference (GRCh37.87) using STAR v2.5.1 [28]. Gene and transcript abundance were quantified using RSEMv1.3.0 [29]. Non-coding genes and those with <10 mapped reads in all samples were excluded. Raw gene counts were analysed using EdgeR [30] and DeSeq2 [31].

### ALL blast RNA-sequencing

IGH-CEBPE translocation BCP-ALL expression data originated from two cohorts. A total of 195 diagnostic cases [32] from Lund University analysed using TCGA RNA sequencing version 2 pipeline quantified by RSEM value and 231 diagnostic [33] cases from the National University of Singapore analysed as per ‘Alignment and Bioinformatic Processing of RNA-Sequencing.’

Gene expression in 2 IGH-CEBPE cases was quantified by *Z*-score calculated from RSEM TPM (transcripts per 10^6^) based on the mean per gene expression of all non-IGH-CEBPE translocated cases in each cohort.

RNA-Seq from 117 diagnostic BCP-ALL cases from TARGET was downloaded from (ftp://caftpd.nci.nih.gov/pub/OCG-DCC/TARGET/ALL/mRNA-Seq/Phase2/L3/expression/BCCA/). Gene expression data were converted to TPM before Spearman correlation analysis in R3.4.2 Blasts were unselected based on molecular subtype.

### SNP arrays

A total of 150 BCP-ALL cases from the Lund cohort with expression data were genotyped using either Illumina Human 1M-duo Infinium BeadChip, HumanOmni1-Quad BeadChip or IlluminaOmni5M BeadChips as previously described [34, 35], and untyped SNPs imputed from the UK10K and 1000 Genomes project reference panels using the Sanger Imputation Server (https://imputation.sanger.ac.uk/).

### Data availability

Raw fastq data of RNA-seq from *CEBPE*-depleted REH cells and CEBPE ChIP-seq from REH has been deposited in the European Genome Phenome archive (https://www.ebi.ac.uk/ega/home EGAS00001002877).

## Results

### Epigenomic profiling of the 14q11.2 locus

**Fig. 1:**
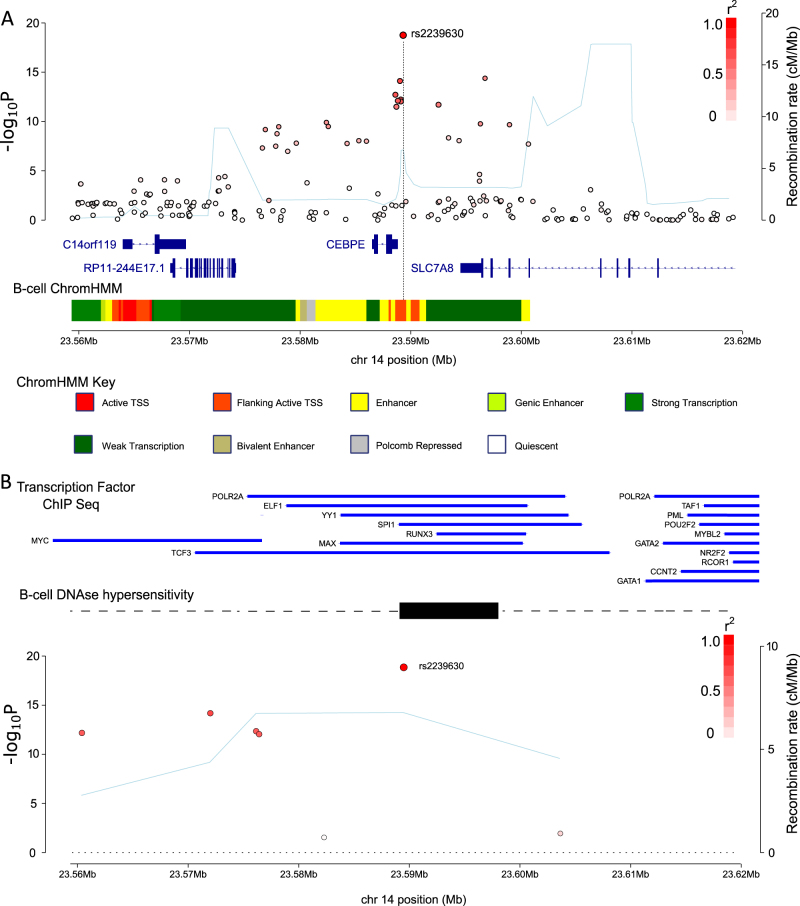
Regional association plot for 14q11.2. **a** and **b** show SNPs (red circles) plotted by GWAS *P*-values (−log_10_, *y*-axis) and location (*x*-axis, GRCh37/hg19). Recombination rate (cM/Mb) light blue line (*y*-axis). SNP colour denotes linkage disequilibrium with the lead SNP, (*r*^2^ = 0, white, *r*^2^ = 1.0, dark red). **a** Association plot annotated with genes from Gencode v27. Multicolour bar shows B-cell chromatin states from ChomHMM. **b** Association plot of SNPs within 1 kb of the lead SNP. Blue lines denote transcription factor ChIP-Seq peaks. Black bar, B-cell DNAse hypersensitivity peak

We reviewed a meta-analysis of UK and German GWASs [8], fine-mapping the 14q11.2 risk locus by imputation. The strongest association was the imputed SNP rs2239630 (*P* = 1.66 × 10^−19^, odds ratio = 1.45, *P*_het_ = 0.38, Fig.1a and Supplementary Table 5). We verified imputation of rs2230630 by sequencing 133 cases. Conditional analysis provided no evidence for an additional independent association. Referencing SNPdb.v150 confirmed imputation captured 100% of variants (minor allele frequency [MAF] > 0.01) within the linkage disequilibrium (LD) block containing rs2239630 (pairwise *r*^2^ ≥ 0.4).

We examined the regulatory potential of SNPs in LD with rs2239630 using chromatin state modelling [36] in primary B-cells, showing lead SNPs reside in an active promoter. Additionally, a region of B-cell DNAse hypersensitivity overlaps rs2239630 (Fig. 1a, b). Encode [37] and Chip Atlas (http://chip-atlas.org/) ChIP-Seq data also revealed binding sites for MAX, YY1, ELF1, SPI1, and TCF3 overlapping rs2239630 (Fig.1b).

### rs2239630 influences *CEBPE* expression and promoter activity

**Fig. 2:**
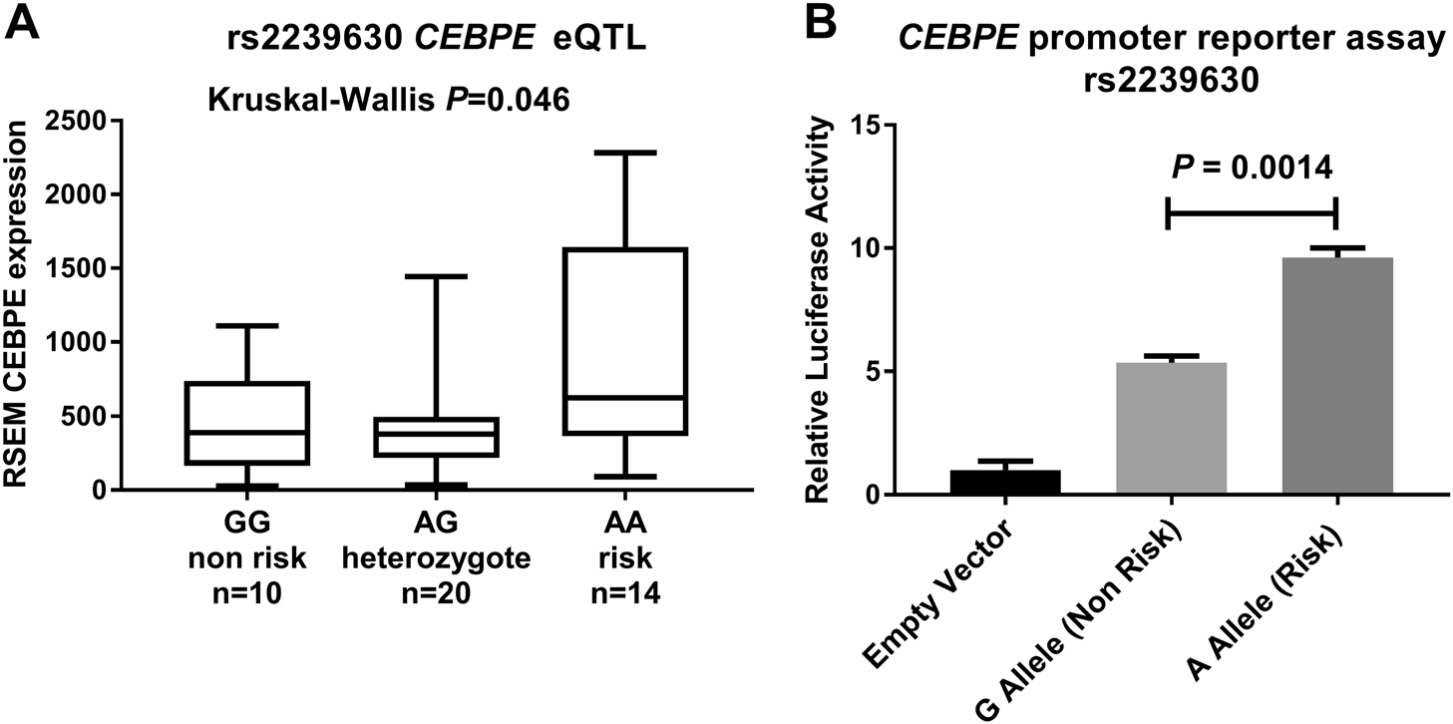
rs2239630 influences *CEBPE* expression and promoter activity. **a** Box (showing interquartile range and median) and whiskers (min–max) plot of *CEBPE* expression (*y*-axis quantified by RSEM) by rs2239630 genotype (*x*-axis) in B-cell ALL blasts (*n* = 44). Kruskal–Wallis *P*-value computed against all genotypes. **b** *CEBPE* promoter reporter assay for alleles of rs2239630. *y*-axis shows normalised luciferase activity relative to empty vector. Data points, mean of three biological replicates ± SEM (Student’s *T*-test). Figures produced in GraphPad Prism 7

We examined whether 14q11.2 risk SNPs influence *CEBPE* expression by performing expression quantitative trait loci analysis (eQTL) in ALL blast cells. Restricting our analysis to chr14 disomic blasts (*n* = 44), the rs2239630-A risk allele was associated with 1.8-fold increased *CEBPE* expression (Kruskal–Wallis *P* = 0.046, Fig. 2a). A similar association was seen in MuTHER (*P* = 0.017) [38] and Blood (*P* = 4 × 10^−4^) [39] lymphoblastoid cell line datasets. To exclude the potential of a looping *cis*-regulatory interaction we examined GM12878 Hi-C data [19]. This revealed an interaction between the 14q11.2 locus and the promoter of *SLC7A8* (Supplementary Fig.1A), however no significant association between rs2239630 genotype and *SLC7A8* expression was seen in either ALL blasts (*P* = 0.14, Supplementary Fig.1B), MuTHER (*P* = 0.31) or blood (*P* > 0.05) eQTLs datasets.

We assessed whether risk SNP genotype influences *CEBPE* promoter activity, performing luciferase reporter assays. REH cells transfected with constructs containing the rs2239630-A risk allele displayed 1.9-fold higher luminescence (*T*-test *P* = 0.0014, Fig. 2b). The same effect was seen in additional ALL cell lines (Supplementary Fig.2). rs2239633 and rs2239632 have been reported to affect *CEBPE* promoter activity in non B-cell lines [40], however no relationship was shown in ALL cell lines (Supplementary Fig.2).

### rs2239630 alleles differentially bind SPI1 and MAX

**Fig. 3:**
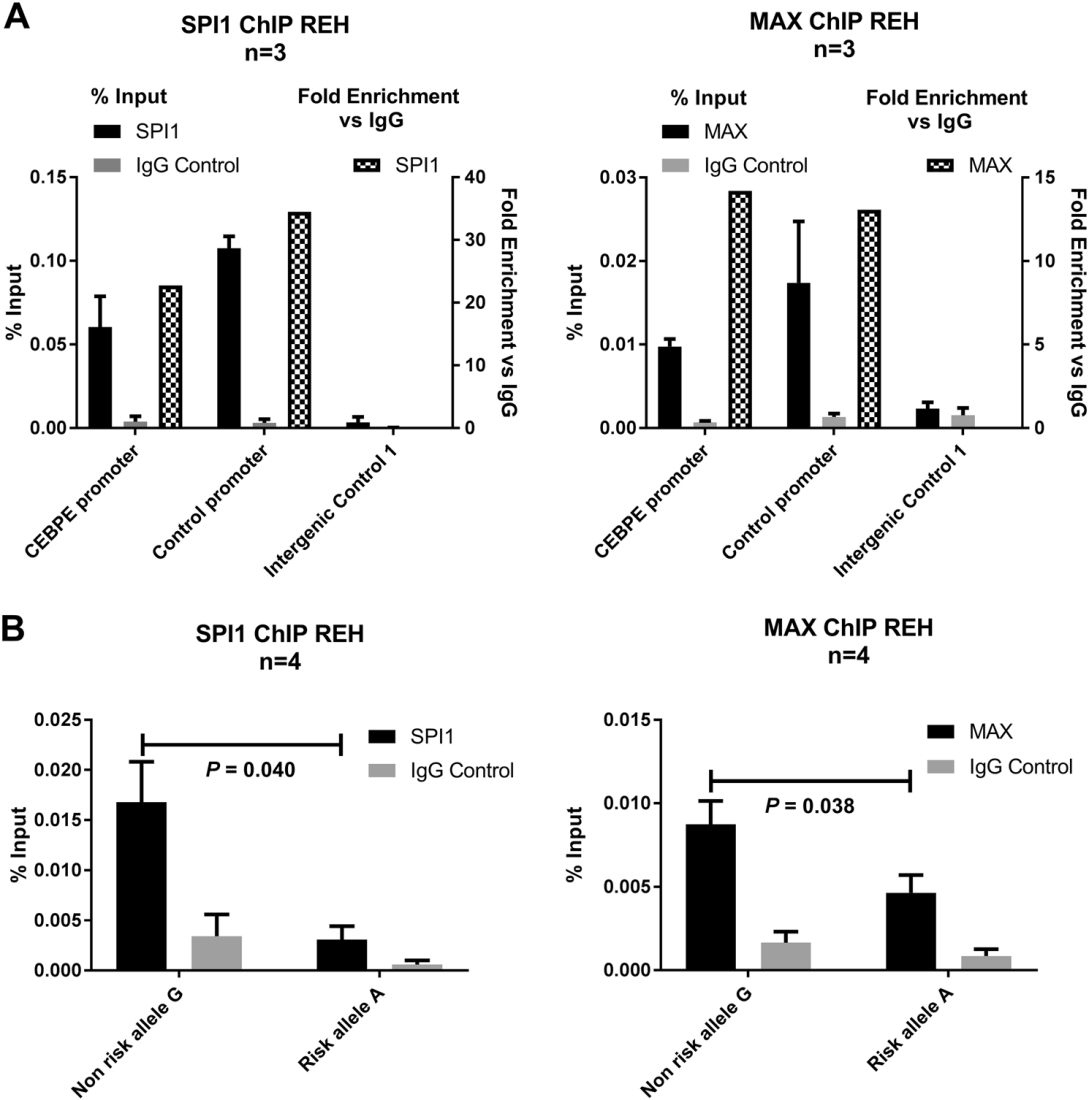
rs2239630 alleles differentially bind SPI1, MAX. **a** ChIP q-PCR of MAX and SPI1 enriched chromatin in REH cells. *x*-axis lists genomic loci of PCR primers used to amplify antibody specific (MAX or SPI1, black bars) or negative control (IgG, grey bars) ChIP samples. Raw signal normalised to input DNA (left *y*-axis). Right *y*-axis shows fold enriched (hashed bars) of antibody specific % input vs IgG control. **b** Allele-specific ChIP q-PCR for MAX and SPI1 in REH

To examine SPI1, MAX, ELF1, YY1, and TCF3 binding at rs2239630 we performed ChIP in REH and NALM6. Only SP1 and MAX bound rs2239630 (Fig. 3a, Supplementary Fig.3A and Supplementary Fig.4). Both REH and NALM6 are heterozygous for rs2239630, allowing allele-specific q-PCR to assess allelic bias of binding. MAX and SPI1 demonstrated greater affinity for rs2230630-G non-risk allele in REH (*T*-test *P* = 0.040 and *P* = 0.038, Fig. 3b) and NALM6 (*T*-test *P* = 0.074 and *P* = 0.074 Supplementary Fig.3B).

### SPI1 regulates *CEBPE* expression

**Fig. 4:**
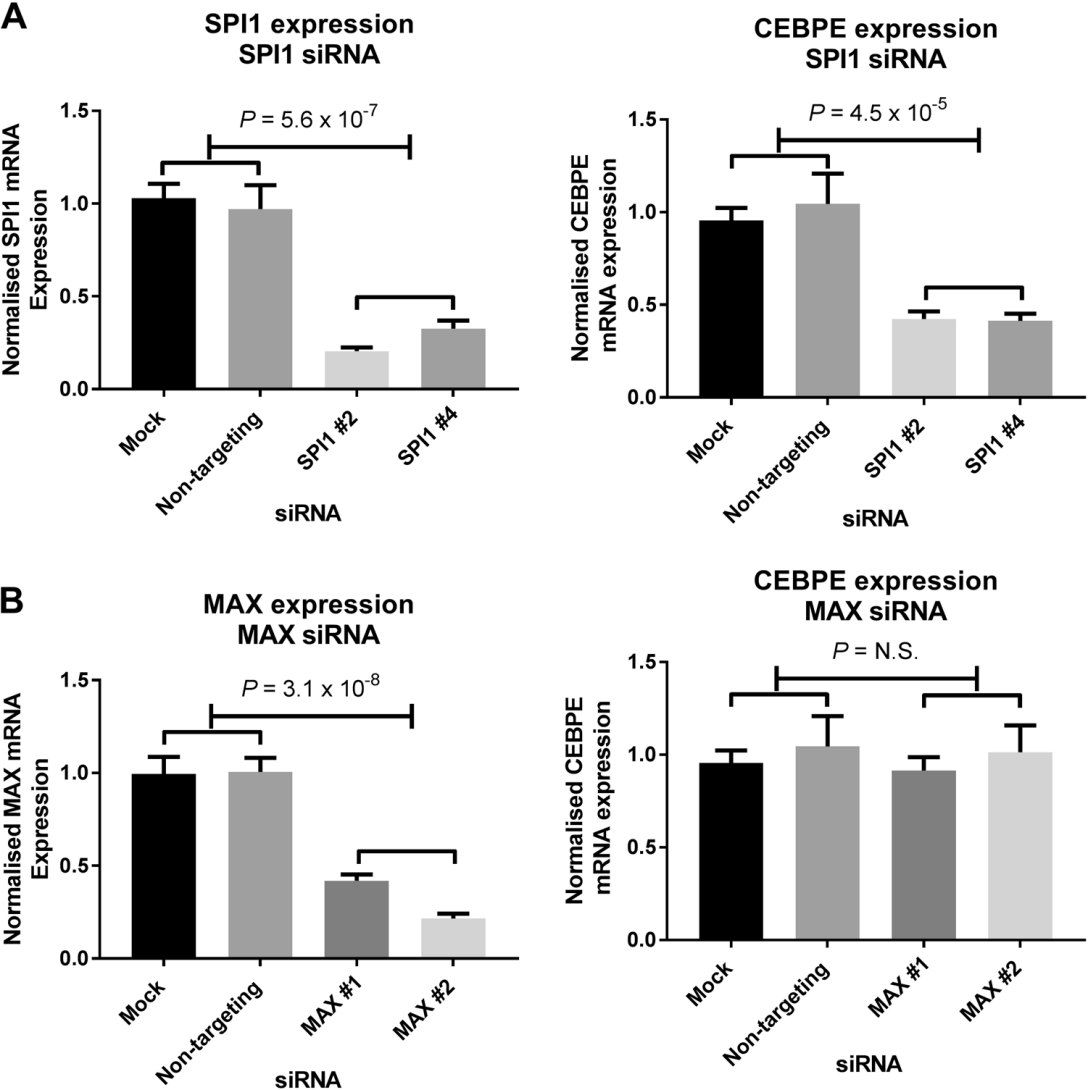
SPI1 but not MAX regulate *CEBPE* expression. **a** qRT-PCR of *SPI1* and *MAX* knockdown in REH cells (*n* = 5). **b** qRT-PCR of *CEBPE* expression in *SPI1* and *MAX* knockdowns (*n* = 5). All data points mean of biological replicates ± SEM. (*T*-test *P*-values). Target gene expression normalised to geometric mean of PPIA and TUBB relative to negative controls (mock and non-targeting siRNA) shown on *y*-axis

We investigated whether differential SPI1 or MAX binding at rs2239630 accounts for eQTL and luciferase assays by performing siRNA knockdowns. Reduced *MAX* expression had no impact on *CEBPE*. Conversely, *SPI1* depletion resulted in decreased *CEBPE* (Fig. 4a, b), a finding inconsistent with rs2239630-A increasing *CEBPE* expression.

### ZNF148 binds the risk allele of rs2239630 and inhibits expression of *CEBPE*

**Fig. 5:**
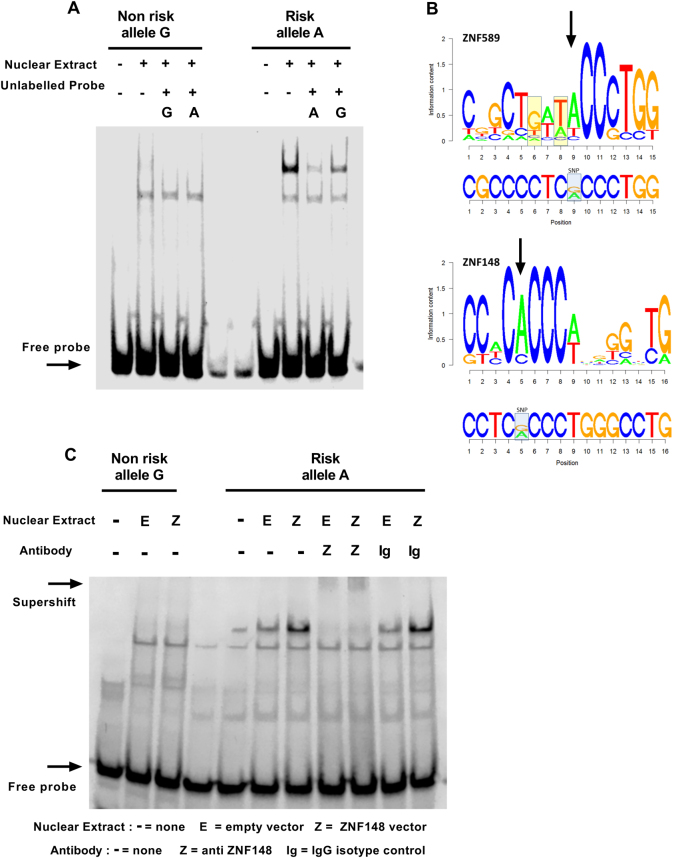
ZNF148 binds the rs2239630 risk allele. **a** EMSA allele-specific probes for rs2239630 incubated with REH nuclear protein. **b** Position weighted matrices for ZNF589 and ZNF148 with corresponding genomic sequence below. Yellow boxes highlight base mismatches. **c** EMSA in REH showing differential allelic binding is caused by ZNF148. Supershift assay performed by addition of anti-ZNF148 antibody or IgG isotype control

**Fig. 6:**
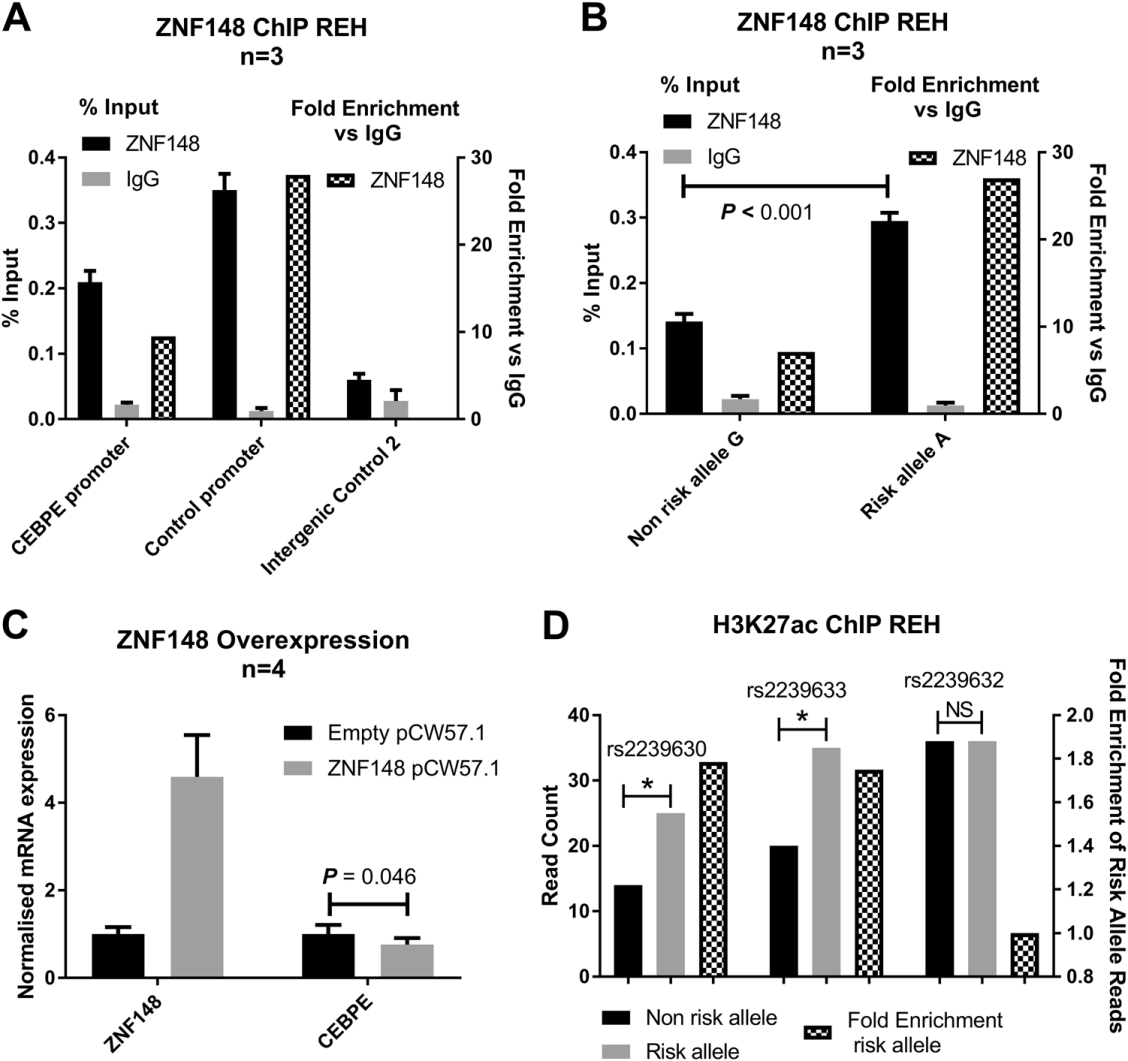
ZNF148 binds the rs2239630 risk allele and represses *CEBPE*. **a** ZNF148 ChIP q-PCR in REH cells. Left *x*-axis raw signal normalised to input DNA, right *y*-axis, fold enrichment (hashed bars) vs IgG control. **b** Allele-specific ChIP q-PCR for ZNF148 in REH. **c** q-RT PCR for *ZNF148* and *CEBPE* mRNA expression in *ZNF148*-overexpressing REH. T-test *P*-values. Target gene expression normalised to geometric mean of PPIA and TUBB, shown relative to empty vector control. Data points, mean of biological replicates ± SEM. (**d**) H3K27ac ChIP-seq in REH. Reads mapping to each allele lead SNPs in the 14q11.2 risk loci are enumerated on the *y*-axis, hashed bars show the enrichment for reads mapping to risk alleles. * binomial *P*-values <0.05

To search for additional allele-specific protein binding at rs2239630 we performed electrophoretic mobility shift assays (EMSA). Greater protein or complex binding was shown for the rs2239630-A allele in REH, SEM, and JURKAT (Fig. 5a and Supplementary Fig.5). We used HaploReg v4.1 [41], RegulomeDB [42], and MotifBreakR [43] to identify motifs disrupted by rs2239630 (Fig. 5b). Using the criteria of preferential affinity for rs2239630-A allele, a predicted strong effect and expression in REH, identified ZNF148 and ZNF589 (Supplementary Table 6). Two mismatches within the motif of ZNF589 overlapping rs2239630 (Fig. 5b) suggested ZNF148 as the most credible candidate. Performing EMSA antibody supershift assays with REH nuclear extract overexpressing ZNF148 identified ZNF148 as the protein preferentially bound to rs2239630-A (Fig. 5c). Allele-specific binding of ZNF148 was confirmed by ChIP-qPCR (Fig. 6a, b). To investigate allele-specific binding of ZNF148 on *CEBPE* we generated inducible *ZNF148-*overexpressing REH cells. Overexpression of *ZNF148* reduced *CEBPE* expression (*T*-test *P* = 0.046, Fig. 6c).

### rs2239630-A is associated with marks of active transcription

Since effects of SPI1 and ZNF148 interaction at rs2239630 were inconsistent with higher risk allele *CEBPE* expression we sought evidence of allele-specific expression (ASE). As it is not possible to directly measure ASE of *CEBPE*, in the absence of a proxy coding SNP for rs2239630, we assayed *CEBPE* promoter H3K27 acetylation. REH H3K27ac ChIP-seq showed significantly higher reads counts mapping to the rs2239630-A risk allele (binomial *P* = 0.027, Fig. 6d), no bias was present in input DNA (binomial *P* = 0.99). Moreover the risk allele bias was higher for rs2239630 than either rs2239632 or rs2239633. These data are consistent with increased transcriptional activity due to allele-specific promoter acetylation.

### Functional and transcriptional profiling of CEBPE

**Fig. 7:**
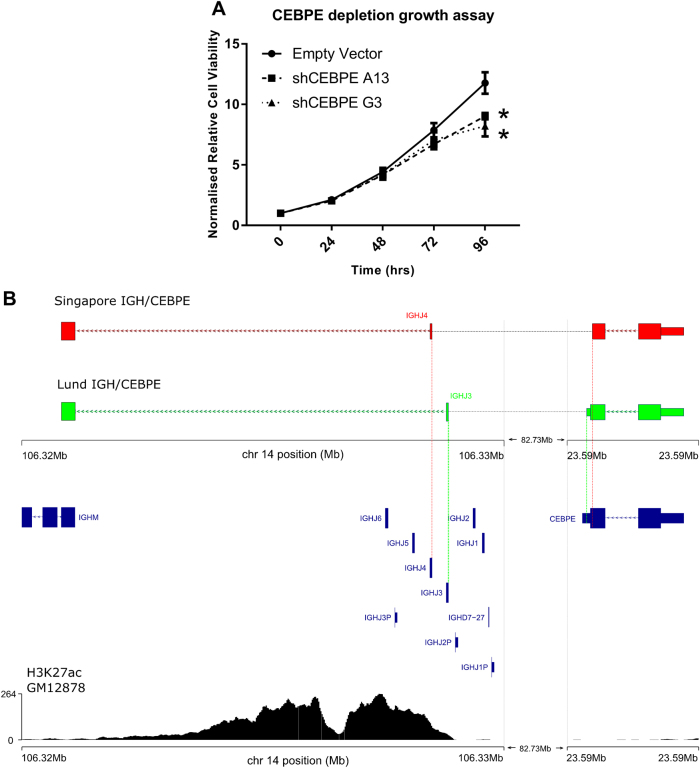
*CEBPE* depletion reduces cell growth. Genomic breakpoints in two IGH-CEBPE ALL cases. **a** Cell viability/growth assay in *CEBPE*-depleted cells. Absorbance (562 nm) normalised to 0 h matched shRNA control shown on *y*-axis. * *T*-test *P-*value <0.05. Data points, mean of four biological replicates ± SEM. **b** Schematic of IGH-CEBPE translocation breakpoints. Upper panel, RNA and Sanger sequencing reads spanning ~83 Mb between breakpoints, denoted by dashed lines. Case-specific breakpoints are denoted by coloured vertical lines. Lower panel, genes mapping to the region and GM12878 H3K27ac ChIP-seq

To investigate CEBPE function in ALL we generated doxycycline inducible *CEBPE* shRNA cell lines, in REH. *CEBPE* knockdown reduced cell growth (Fig. 7a), but did not affect cell cycle or apoptosis (Supplementary Fig.6A). *CEBPE-*depleted cells did not show differential apoptosis either alone or in combination with cisplatin or TNFα (Supplementary Fig.6B-D).

To identify genes and pathways regulated by CEBPE, we performed ChIP-Seq and RNA-Seq in *CEBPE*-depleted cells. Peak calling of ChIP-Seq data identified 313 enriched loci, 83 mapping within 1 kb of a transcription start site (TSS) (Supplementary Table 7). We identified enriched motifs using HOMER 4.9.1, revealing the CEBPE consensus motif as TTGCGCAA (*P* = 1 × 10^−150^) (Supplementary Table 8). Protein-coding genes showing the highest promoter enrichment were *GAS7*, *SPRY4*, *ANKRD13D*, *TFEB*, and *ADGB*. Pathways enriched for putative CEBPE-regulated genes were interrogated using REACTOME [44] and PANTHER [45]. Analyses were consistent with CEBPE binding sites being enriched in the promoters of genes involved in precursor metabolites and energy biogenesis (*P*_REACTOME_ = 5.8 × 10^−3^, *P*_Panther_ = 4.2 × 10^−3^) and respiration/electron transport (*P*_REACTOME_ = 0.01, *P*_Panther_ = 0.067), which may contribute to reduced metabolic activity and growth of *CEBPE* depleted cells.

We identified genes regulated by *CEBPE* by performing RNA-Seq in *CEBPE*-depleted cells (Supplementary Table 9). Consistent with a role in granulocyte development, *CEBPE* positively regulated *MPO* and *PLD1*, genes of B-cell relevance included *IL7R*, *PRAME*, *BCL2*, and *RASSF4*, each positively regulated. To identify differentially expressed genes directly regulated by *CEBPE* we mapped ChIP-Seq peaks and gene TSS using Hi-C data in lymphoblastoid and embryonic stem cells [46]. Findings were consistent with direct regulation for 13 of 73 top differentially expressed genes. Hi-C contacts between ChIP-Seq peaks and TSS were seen for 10 of 73 genes, including *BCL2*, *MPO*, and *FAM69C* (Supplementary Table 9). The resolution of Hi-C and proximity of *UGT3A2* and *CDH12* TSS with ChIP-Seq peaks precluded demonstration of an interaction. Finally, the promoter of *C4orf32* bound *CEBPE* directly.

To evaluate the similarity of *CEBPE*-regulated genes in *CEBPE*-depleted and BCP-ALL blast cells we examined RNA-Seq from 117 diagnostic cases from TARGET [47]. First, we identified *CEBPE*-correlated genes, restricting our analysis to those correlated at *P* < 5 × 10^−4^, a significant enrichment in differentially expressed genes from *CEBPE*-depleted cells was seen (binomial *P* = 5.4 × 10^−4^). We next examined the similarity of *CEBPE*-correlated gene profiles from two IGH-CEBPE translocated BCP-ALL cases and 117 ALL blasts. *CEBPE* was highly over-expressed in IGH-CEBPE translocated cases (mean *Z*-score = 4.6) and the top 150 differentially expressed genes were highly enriched for *CEBPE*-correlated genes in the 117 BCP-ALL cases (Binomial *P* = 2.9 × 10^−7^).

### Mapping IGH-CEBPE breakpoints

To explore the integrity of the *CEBPE* transcript associated with t(14:14) IGH-CEBPE ALL, we mapped chr14 breakpoints by RNA and Sanger sequencing of genomic DNA. In contrast to the breakpoints in one previously reported IGH-CEBPE case [13] we observed breakpoints in the 3′ of *CEBPE* and the 5′ of IGHJ transcripts in both cases (Fig. 7b and Supplementary Figure 7 and 8). Although in one case breakpoints truncated the terminal 13 amino acids of *CEBPE* this affected a region of no known function suggesting no functional impact, and in a second case the coding sequence was unaffected.

The locus upstream of IGHJ breakpoints contains extensive H3K27ac, associated with enhancer activity, consistent with this region driving increased *CEBPE* expression (Fig. 7). The position breakpoints at IGHJ implicate aberrant RAG1 and/or RAG2 somatic VDJ recombination in their formation [48]. However, examination downstream of *CEBPE* breakpoints revealed only consensus RAG hexamer sequence, and not the nonamer sequence required for canonical RAG activity.

## Discussion

Our data provide a plausible mechanism of increased ALL risk at 14q11.2, driven by increased *CEBPE* expression mediated via the rs2239630-A risk allele. Epigenetic, eQTL and luciferase data suggest differential promoter activity conferred by rs2239630 results in allele-specific expression of *CEBPE*.

We identified allele-specific interaction of SPI1 and ZNF148 at rs2239630. The regulatory impact of which is to attenuate increased *CEBPE* expression associated with rs2239630-A. This suggests that modulation of expression due to allele-specific binding of transcription factors is complex and the aggregate of multiple interacting proteins.

Previously, Wiemels et al. [49] proposed rs2239635 as the 14q11 functional variant for ALL. rs2239635, is weakly correlated with rs2239630 (*r*^2^ = 0.52) mapping to the 5′-UTR of *CEBPE* (Supplementary Figure 9). In contrast to rs2239630, neither GTEx v7, Blood [39] nor MuTHER [38] datasets showed a relationship between rs2239635 genotype and *CEBPE* expression and RNA-Seq data in chr14 disomic ALL blasts heterozygous for rs2239635 showed no allele bias (*P* = 0.92, *n* = 20). The authors proposed IKZF1 binding at rs2239635 based on low ChIP enrichment (<2-fold) in bone marrow, which contains a low proportion of B-cell lineages. An analysis of eight B-cell ChIP-Seq datasets in five B-cell types, including precursors, showed no evidence of IKZF1 binding rs2239635 [50–53]. Additionally in silico data, cited by the authors, predicts only a minimal impact of rs2239635 on IKZF1 binding despite apparent abolition of risk allele occupancy (Supplementary Table 10). Furthermore, data from ENCODE suggest rs2239635 is unbound by transcription factors and possesses reduced marks of active transcription relative to rs2239630.

*CEBPE* is a member of the CEBP transcription factor family primarily expressed during granulocyte differentiation. Homozygous mutations in *CEBPE* cause congenital-specific granule deficiency [54] and mice with *CEBPE* disruption have myelopoietic defects [55]. However BCP-ALL featuring IGH translocations involving *CEBP* genes establishes their role in B-cell oncogenesis [13].

We have shown *CEBPE* depletion reduces growth in ALL cells. Transcriptional profiling of *CEBPE*-depleted cells demonstrates that it regulates the expression of genes with a role in B-cell development (*IL7R* [56]), apoptosis inhibition (*BCL2* [57]), methotrexate resistance (*RASSF4* [58]) and cell survival (*PRAME* [59]), identifying potential mediators of disease development.

IL7 signalling is required for adult bone marrow B-cell production, where it increases B-cell differentiation, proliferation, and survival [60]. Correspondingly, IL7R−/− mice do not respond to IL7 and have severely reduced B- and T-cell counts [61]; whereas, transgenic IL7-overexpressing mice develop lymphoproliferative disorders of B- and T-cell compartments [62].

BCL2 (B-cell lymphoma 2) expression is elevated in many tumours, including ALL [63, 64], and has been associated with an adverse prognosis. Correspondingly, BCL2 inhibition induces apoptosis in a number of ALL cell lines [57], but not REH, this response has been associated with serine 70 phosphorylation of BCL2 [65], a mark lacking in these cells [66]. BCL2 is currently under investigation as a target for therapy [67].

PRAME (preferentially expressed antigen in melanoma) expression is elevated in ALL [68] and other malignancies [59]. PRAME antagonises retinoic acid signalling promoting cell survival [69]. It encodes an antigen recognised by autologous cytolytic T lymphocytes and has been suggested as a target for immunotherapy.

Inherited variation and expression of *RASSF4* are associated with accumulation of methotrexate polyglutamates [58]. Methotrexate is one of the main components of ALL treatment and conversion of the pro-drug to a polyglutamated form is required for efficacy.

Taken together this suggests *CEBPE* influences the expression of genes potentially contributing to various stages of disease development and progression.

Comparing the expression profiles of *CEBPE*-depleted cells to BCP-ALL blasts we find *CEBPE* expression affects similar transcriptional programs in both. We also compare *CEBPE*-regulated genes in two cases with IGH-CEBPE translocation with BCP-ALL blasts, again demonstrating significant similarities suggesting that the mechanism by which *CEBPE* drives leukemogenesis is the same for 14q11.2 associated and translocated ALL.

In conclusion, we have shown increased *CEBPE* expression in ALL patients carrying the rs2239630-A risk allele, and identified genes, involved in B-cell development and apoptosis, via which *CEBPE* may influence the risk of disease, thus providing a mechanistic basis for the 14q11.2 risk association for ALL. Further functional studies, however, are required to fully decipher the biological basis of differential *CEPBE* expression on ALL oncogenesis.

## Electronic supplementary material

Supplementary Material
