# Supplementary material for: Genetic predisposition to B-cell acute lymphoblastic leukemia at 14q11.2 is mediated by a CEBPE promoter polymorphism
Source: Leukemia. 2018 Jul 6;33(1):1–14. doi: 10.1038/s41375-018-0184-z (PMC6327050; doi:10.1038/s41375-018-0184-z)
Supplement: Supplementary file 1 — Supplementary Material [file 41375_2018_184_MOESM1_ESM.docx]

**Genetic predisposition to B-cell acute lymphoblastic leukemia at 14q11.2 is mediated by a *CEBPE* promoter polymorphism**

James B Studd^1*^, Minjun Yang^2^, Zhenhua Li^3^, Jayaram Vijayakrishnan^1^, Yi Lu^3^, Allen Eng-Juh Yeoh^3,4^, Kajsa Paulsson^2^, Richard S Houlston ^1,5^

1. Division of Genetics and Epidemiology, The Institute of Cancer Research, London, SM2 5NG, United Kingdom.

2. Department of Laboratory Medicine, Division of Clinical Genetics, Lund University, 221 85 Lund, Sweden.

3. Centre for Translational Research in Acute Leukaemia, Department of Paediatrics, Yong Loo Lin School of Medicine, National University of Singapore, Singapore.

4. Viva-University Children’s Cancer Centre, Khoo Teck Puat-National University Children’s Medical Institute, National University Hospital, National University Health System, Singapore, Singapore.

5. Division of Molecular Pathology, The Institute of Cancer Research, London, SM2 5NG, United Kingdom.

**Supplementary Methods and Material**

**Electrophoretic Mobility Shift Assay**

Nuclear protein was extracted using the NE-PER Nuclear and Cytoplasmic Extraction kit (Thermo Fisher Scientific). 5’ fluorescently labelled (DayLight 682nM) and unlabelled complementary oligonucleotides (Eurofins Genomics) flanking rs2239630 (5′-AGGCTGGTGCTTCGCCCCTC[A/G]CCCTGGGCCTGAGGCTCTGC-3′) were annealed. Binding reaction contained 50fmol labelled probe DNA, binding buffer (10mM Tris, 30mM KCl, 2mM DTT, 2.5% glycerol, 0.01mg/ml BSA [pH7.5]), 1μg polydI-dC [Sigma-Aldrich], and 10μg protein in 20µl. Reactions were incubated in the dark for 30mins at RT. Competition assays were performed by adding 100-fold molar excess of unlabelled probes. Super-shift assays were performed by the addition of 2µg anti-ZNF148 (Atlas Antibodies HPA001656) or IgG isotype control (AbCam ab37415) to binding reactions and incubating for additional 30 mins at 4^o^C prior to probe addition. Reactions were resolved by electrophoresis on a 6% DNA retardation gel (Life Technologies) in 0.5×Tris-borate-EDTA (TBE) at 4°C and imaged (Odyssey Fc Infrared Imaging System, LI-COR Biosciences).

**Plasmid Construction and Luciferase Assays**

The *CEBPE* promoter region containing rs2239630, rs2239632 and rs2239633 and *ZNF148* coding regions were amplified from genomic DNA from the CEU LCL NA11830 (*CEBPE* promoter) and cDNA from REH (*CEBPE* and *ZNF148* coding regions) using Phusion Taq polymerase (NEB) (primers in **Supplementary Table 1).** Gel purified PCR-products (Qiagen) were A-tailed using 2U Thermprime DNA polymerase (ThermoFisher Scientific) and 200µM dATP (30mins at 70^o^C) and cloned into pCR/8/GW/TOPO (ThermoFisher Scientific). Bacterial colonies were picked, cultured and DNA purified using Qiagen Mini-prep Kit. For promoter sequences alternate SNP alleles were generated using site-directed mutagenesis (Quick Change XL, Agilent; primers in **Supplementary Table 1)**. Inserts were transferred into a Gateway compatible pGL3 Promoter Vector (Promega) or pCW57.1 doxycycline inducible expression vector for promoter and coding sequences respectively, using Gateway LR Clonase II (ThermoFisher Scientific). Constructs were verified by Sanger sequencing (primers in **Supplementary Table 1)**. For luciferase assays cells were electroporated using a Nucleofector 2B (Lonza). 4x10^6^ (REH), 2x10^6^ (NALM6, SEM) and 1x10^6^ (Jurkat) cells were transfected with 2.5µg of pGL3 promoter and 50ng of pRL-SV40 (Promega, Madison), in 100µl of solution R (REH), V (SEM and Jurkat) or T (Nalm6) using either program X-01 (REH and Jurkat), T-02 (SEM) and or C-05 (NALM6). After 24hrs promoter activity was assayed using a Dual-Luciferase assay (Promega) on a Fluoroskan Ascent FL (Labsystems).

**Statistics**

Datasets on which Student’s t-tests were performed are normally distributed. All Student’s t-tests were unpaired ‘2-sided’ and assume unequal variance. For all luciferase and qRT data each biological replicates are the mean of 3 technical replicates.

**Supplementary Table 1: Primer sequences**

| **Primer name** | **Assay** | **Sequence** |
| --- | --- | --- |
| CEBPE promoter fwd | Luciferase cloning | CTGATGCTTCTGGGAATGCCT |
| CEBPE promoter rev | Luciferase cloning | AGCAGTACCAGAGCAGGTGT |
| rs2239630 fwd | SDM | CAGGCCCAGGGCGAGGGGCGAAG |
| rs2239630 rev | SDM | CTTCGCCCCTCGCCCTGGGCCTG |
| rs2239632 fwd | SDM | TGGAAACGCACTAACATTTGGGCTATTGCACAGCT |
| rs2239632 rev | SDM | AGCTGTGCAATAGCCCAAATGTTAGTGCGTTTCCA |
| rs2239633 fwd | SDM | CACCACGCAGGCTCGTGTGTAGAGCTTGTTC |
| rs2239633 rev | SDM | GAACAAGCTCTACACACGAGCCTGCGTGGTG |
| CEBPE promoter fwd | Sequencing | AGTGTAGGCCAGGGTTTGTT |
| CEBPE promoter rev | Sequencing | TGGGTCTACTTCCTCTTGTGA |
| CEBPE promoter fwd | Sequencing | GTCAAGGGAGGAGGTGAGAC |
| CEBPE promoter rev | Sequencing | ACAGGAAACCAGAGGCAGAG |
| rs2239630 genotyping | Sequencing | CCCTCGGAGTAGACAGCCAT |
| rs2239630 genotyping | Sequencing | GTAGACCCAAGAGACACGCA |
| CEBPE gene fwd | Gene cloning | CCAGGTCAGGAGGAGGTAGA |
| CEBPE gene rev | Gene cloning | GGGGTCCGCAGAGTTAGG |
| ZNF148 coding fwd | Gene cloning | ACCTTAGTCTCGGGCAGTTA |
| ZNF148 coding rev | Gene cloning | CAGCACTCCATTTACACAGAGT |
| IGHJ4 fwd | CEBPE/IGH breakpoint | GACGGTGACCAGGGTTCC |
| CEBPE exon 2 rev | CEBPE/IGH breakpoint | GCTGGAGTACATGGCAGAGA |
| IGHJ4 upstream fwd | CEBPE/IGH breakpoint | GGAGACCCAGCACGCTTATT |
| IGHJ4 upstream mk2 fwd | CEBPE/IGH breakpoint | CTTGCCCCTCGTCTGTGT |
| CEBPE exon 2 mk 2 rev | CEBPE/IGH breakpoint | GTACATGGCAGAGAACGAGC |

SDM: site directed mutagenesis

Breakpoint PCR conditions: 95oC – 5min; 35 cycles of (95^o^C – 30s; 60^o^C – 30s; 72^o^C – 1min); 72^o^C – 5min.

**Supplementary Table 2: ChIP-qRT and RT-PCR primer sequences**

| CEBPE promoter fwd | | ChIP-qRT | | TGGCTCCAGCACTTCTCTCT | |
| --- | --- | --- | --- | --- | --- |
| CEBPE promoter rev | | ChIP-qRT | | CAGGCACAGGAAACCAGAGG | |
| SPI1 control (ITGAM) fwd | | ChIP-qRT | | AGACTTCTGCCTCCTACTTCTC | |
| SPI1 control (ITGAM) rev | | ChIP-qRT | | AGAACCTGGAAGGAGGTGAA | |
| YY1 control (CXXC1) fwd | | ChIP-qRT | | CCTCGACGTACAGGCAACAT | |
| YY1 control (CXXC1) rev | | ChIP-qRT | | GAATTCGACCCACGTACGGA | |
| ELF1 control rev (ZNF169) fwd | | ChIP-qRT | | GTAGTGTAGTTCCGTGCCCA | |
| ELF1 control (ZNF169) rev | | ChIP-qRT | | TGAAAGGACACTCACCACGC | |
| MAX control (CDK6) fwd | | ChIP-qRT | | GGGGCTGCGAGTGTCAG | |
| MAX control (CDK6) rev | | ChIP-qRT | | CCGGAGAGAGTGCTGGTAAC | |
| Intergenic Control 1 fwd | | ChIP-qRT | | AAAGCCGCTCAACTACATGG | |
| Intergenic control 1 rev | | ChIP-qRT | | TGCTTTGAATGCGTCCCAGAG | |
| ZNF148 control (VIM) fwd | | ChIP-qRT | | TGAAGTAACGGGACCATGCC | |
| ZNF148 control (VIM) rev | | ChIP-qRT | | AGCCAATAGGGACCTAGCGA | |
| E2A control (MRPL34) fwd | | ChIP-qRT | | CCTCTGCCGGTCAATAGGAG | |
| E2A control (MRPL34) rev | | ChIP-qRT | | CGTGCGCACAAGGCATTATC | |
| Intergenic control 2 fwd | | ChIP-qRT | | ATGTCAGGCCCATGAACGAT | |
| Intergenic control 2 rev | | ChIP-qRT | | CATTCATGGAGTCCAGGCTT | |
| CEBPE fwd | | RT-PCR | | CTCCGATCTCTTTGCCGTGAA | |
| CEBPE rev | | RT-PCR | | CCGAAGGTATGTGGAGGGTAG | |
| ZNF148 fwd | | RT-PCR | | CAGGACAATGGTTGTAATGGGT | |
| ZNF148 rev | | RT-PCR | | GGTGAGGCATACTTCGATCTTGA | |
| MAX fwd | | RT-PCR | | TGCACTGGAACGAAAACGTAG | |
| MAX rev | | RT-PCR | | GTCGTCAATATCTTGCTGGTGT | |
| SPI1 fwd | | RT-PCR | | GTGCCCTATGACACGGATCTA | |
| SPI1 rev | | RT-PCR | | AGTCCCAGTAATGGTCGCTAT | |
| PPIA fwd | | RT-PCR | | CTGCACTGCCAAGACTGA | |
| PPIA rev | | RT-PCR | | GCCATTCCTGGACCCAAA | |
| TBP fwd | | RT-PCR | | TGCACAGGAGCCAAGAGTGAA | |
| TBP rev | | RT-PCR | | CACATCACAGCTCCCCACCA | |
| G6PD fwd | | RT-PCR | | GATGCCTTCCATCAGTCGGA | |
| G6PD rev | | RT-PCR | | GCTCACTCTGTTTGCGGATG | |
| TUBβ fwd | | RT-PCR | | AGCAGCGTGAGTTTGAGAGC | |
| TUBβ rev | | RT-PCR | | AGACTGCCTGGCATTGTCC | |

Q-RT-PCR cycling conditions;

950C – 10 min; 40 cycles of - (95^0^C – 10 sec; 58^0^C – 20 sec)

**Supplementary Table 3: siRNA sequences**

| SPI1 #2 | Sense 5’-3’ | UCC AUU UUG CAC GCC UGU AAC AUC CAG |
| --- | --- | --- |
| SPI1 #2 | Anti-sense 5’-3’ | GGA UGU UAC AGG CGU GCA AAA UG dGdA |
| SPI1 #4 | Sense 5’-3’ | UAC CAA CGC CAA ACG CAC GAG UAU U |
| SPI1 #4 | Anti-sense 5’-3’ | AAU ACU CGU GCG UUU GGC GUU GGU A |
| MAX1 #1 | Sense 5’-3’ | UGC CGA AGA AUU GUC UUG CAA GUU A |
| MAX1 #1 | Anti-sense 5’-3’ | UAA CUU GCA AGA CAA UUC UUC GGC A |
| MAX1 #2 | Sense 5’-3’ | UUC ACU CAG AUU CAA AUU UAA GUA GCA |
| MAX1 #2 | Anti-sense 5’-3’ | CUA CUU AAA UUU GAA UCU GAG UG dAdA |
| Non Targeting | Sense 5’-3’ | AGG UAG UGU AAU CGC CUU GCU UCA UGC |
| Non Targeting | Anti-sense 5’-3’ | GCA UGA AGC AAG GCG AUU ACA CUA CCU |

**Supplementary Table 4: Summary of RNA-sequencing quality control metrics**

| Sample | RIN | Tru Seq Index / Lane | Number of reads pre filtering paired | Number of reads post filtering paired | % Uniquely aligned reads |
| --- | --- | --- | --- | --- | --- |
| Empty  rep 1 | 10 | 2 / 1 | 25052986 | 24448052 | 88.36% |
| Non-Silencing rep 1 | 10 | 9 / 1 | 24778156 | 24092195 | 87.88% |
| CEBPE A13  Rep 1 | 9 | 10 / 1 | 30258569 | 28893284 | 83.28% |
| CEBPE G3 rep 1 | 7.7 | 11 / 1 | 45463124 | 42553493 | 80.61% |
| Empty  rep 2 | 10 | 5 / 1 | 48107508 | 47264411 | 86.93% |
| Non-Silencing rep 2 | 10 | 7 / 1 | 46335707 | 45898668 | 86.71% |
| CEBPE A13  rep 2 | 10 | 5 / 2 | 40149816 | 39785329 | 87.73% |
| CEBPE G3 rep 2 | 10 | 7 / 2 | 23243914 | 23021508 | 87.55% |
| Empty  rep 3 | 10 | 2 / 2 | 37463983 | 37196231 | 87.19% |
| Non-Silencing rep 3 | 10 | 9 / 2 | 38526647 | 38012150 | 87.36% |
| CEBPE A13  rep 3 | 10 | 10 / 2 | 39032044 | 38426728 | 87.40% |
| CEBPE G3 rep 3 | 10 | 11 / 2 | 37300281 | 36994649 | 88.28% |

Summary of RNA-sequencing quality control metrics. RIN (RNA integrity number).

**Supplementary Table 5: Lead SNPs at the chromosome 14 risk locus.**

| rsID | Chr14 pos (hg19) | Risk Allele | Non-Risk Allele | MAF | *P*-value | Odds Ratio | r^2^ |
| --- | --- | --- | --- | --- | --- | --- | --- |
| rs2239630 | 23589349 | A | G | 0.3863 | 1.66E-19 | 1.45 | 1 |
| rs11625112 | 23596740 | A | G | 0.3882 | 3.98E-15 | 1.38 | 0.46 |
| rs2239633 | **23589057** | **G** | **A** | **0.7131** | **7.50E-15** | **1.37** | **0.73** |
| rs12434881 | 23588642 | G | A | 0.9401 | 1.85E-13 | 1.35 | 0.67 |
| rs2239632 | 23589127 | C | A | 0.4448 | 5.68E-13 | 1.34 | 0.65 |
| rs2239634 | 23588863 | T | A | 0.4493 | 7.50E-13 | 1.33 | 0.65 |
| rs2239631 | 23589130 | G | A | 0.4576 | 8.98E-13 | 1.33 | 0.65 |
| rs67260899 | 23592539 | CAGGGA | C | 0.8454 | 1.89E-12 | 1.34 | 0.51 |
| rs2239635 | 23588731 | G | C | 0.1263 | 3.07E-12 | 1.36 | 0.52 |

Lead SNPs in the 14q11.2 ALL risk locus displayed in *P*-value order (fixed effects meta-analysis of logistic regression *P*-value). MAF (minor allele frequency). r^2^ linkage disequilibrium to lead SNP rs2239630 calculated from UK10K and European 1000 Genome individuals. Underlined SNPs typed in German GWAS, **bold** SNPs are typed in the UK GWAS, the remainder being imputed.

**Supplementary Table 6: Candidate Transcription factors binding rs2239630.**

| **Predicted Motif** | **Greater affinity** | **Effect** | **Expressed in REH** |
| --- | --- | --- | --- |
| ZNF148 | A | strong | Y |
| ZNF589 | A | strong | Y |
| KLF1 | A | strong | N |
| TFAP2 | G | strong | na |
| KLF7 | A | weak | na |
| BDP1 | A | weak | na |
| ERS1 | A | weak | na |
| MZF1 | A | weak | na |
| REST | G | weak | na |
| ZFX | G | weak | na |

List of candidate transcription factors binding to rs2239630 extracted from HaploReg v4.1 (1), RegulomeDB (2) and MotifBreakR (3). Expression in REH based on data extracted from human protein atlas (https://www.proteinatlas.org/).

**Supplementary Table 7: Putative CEBPE regulated genes from ChIP-Seq**

| **ChIP peak** | | | **Gene Information** | |
| --- | --- | --- | --- | --- |
| **chr** | **ChIP peak pos** | **Fold background** | **TSS pos** | **Gene Symbol** |
| 19 | 11435156 | 40.96166 | 11435117 | CTC-510F12.4 |
| 17 | 10018739 | 27.58781 | 10017870 | GAS7 |
| 5 | 1.42E+08 | 27.3947 | 1.42E+08 | SPRY4 |
| 11 | 67056014 | 26.14817 | 67056018 | ANKRD13D |
| 6 | 41691340 | 25.71634 | 41691464 | TFEB |
| 6 | 1.47E+08 | 24.1191 | 1.47E+08 | ADGB |
| 19 | 55084346 | 22.56449 | 55084387 | LILRA2 |
| 5 | 1.7E+08 | 22.56449 | 1.7E+08 | CTB-114C7.3 |
| 6 | 30749810 | 22.56449 | 30749735 | HCG20 |
| 2 | 1.2E+08 | 22.52301 | 1.2E+08 | C2orf76 |
| 8 | 8860242 | 21.77501 | 8860314 | ERI1 |
| 3 | 45883680 | 21.48639 | 45883658 | LZTFL1 |
| 12 | 1.23E+08 | 19.50921 | 1.23E+08 | AC156455.1 |
| 1 | 1.61E+08 | 17.38484 | 1.61E+08 | DEDD |
| 20 | 34330355 | 16.50551 | 34330234 | RBM39 |
| 12 | 6451246 | 15.98727 | 6451251 | TNFRSF1A |
| 10 | 1.13E+08 | 15.81635 | 1.13E+08 | SHOC2 |
| 1 | 1.1E+08 | 15.61257 | 1.1E+08 | KIAA1324 |
| 5 | 52856391 | 14.61363 | 52856462 | NDUFS4 |
| 6 | 86353920 | 14.61363 | 86353510 | SYNCRIP |
| 12 | 66563080 | 13.63061 | 66563291 | TMBIM4 |
| 17 | 66342817 | 13.63061 | 66342888 | ARSG |
| 3 | 1.84E+08 | 13.63061 | 1.84E+08 | ALG3 |
| 6 | 1.26E+08 | 13.63061 | 1.26E+08 | NCOA7 |
| 16 | 4103748 | 13.17265 | 4104191 | ADCY9 |
| 1 | 2.4E+08 | 12.58844 | 2.4E+08 | CHRM3 |
| 17 | 37310775 | 12.28933 | 37310647 | PLXDC1 |
| 14 | 61943715 | 12.23594 | 61943885 | PRKCH |
| 20 | 17663377 | 12.23594 | 17662939 | RRBP1 |
| 16 | 22308540 | 12.12865 | 22308733 | POLR3E |
| MT | 793 | 11.79086 | 648 | MT-RNR1 |
| 14 | 35873071 | 11.65259 | 35873346 | NFKBIA |
| 4 | 8160745 | 11.51565 | 8160558 | ABLIM2 |
| 16 | 84651866 | 11.17317 | 84651684 | COTL1 |
| 14 | 21077497 | 10.88053 | 21077954 | RNASE11 |
| 11 | 14542009 | 10.87712 | 14541991 | PSMA1 |
| 17 | 75437622 | 10.87712 | 75438191 | Y_RNA |
| 12 | 54069959 | 10.81158 | 54069899 | ATP5G2 |
| 11 | 82868036 | 10.68054 | 82868030 | PCF11 |
| 16 | 87812897 | 10.18739 | 87812980 | RP4-536B24.4 |
| 12 | 98898044 | 9.82998 | 98897633 | RP11-181C3.1 |
| 1 | 1711996 | 9.43057 | 1711896 | NADK |
| 10 | 16859780 | 9.41668 | 16859527 | RSU1 |
| 17 | 8022535 | 8.66016 | 8022365 | ALOXE3 |
| 11 | 45939488 | 8.21739 | 45939418 | PEX16 |
| 3 | 49843055 | 8.21739 | 49843678 | MIR5193 |
| 9 | 1.31E+08 | 8.21739 | 1.31E+08 | NAIF1 |
| 2 | 1.78E+08 | 8.18605 | 1.78E+08 | NFE2L2 |
| 12 | 22403953 | 8.09194 | 22403721 | ST8SIA1 |
| 16 | 87417686 | 7.80685 | 87417662 | MAP1LC3B |
| 15 | 89878305 | 7.51355 | 89878284 | RP11-217B1.2 |
| 1 | 23857894 | 7.23158 | 23857712 | E2F2 |
| MT | 16117 | 7.17557 | 16023 | MT-TP |
| 1 | 6320209 | 6.94993 | 6321035 | GPR153 |
| 1 | 1.61E+08 | 6.94993 | 1.61E+08 | NDUFS2 |
| 16 | 3105242 | 6.94993 | 3104776 | RP11-473M20.7 |
| X | 1656459 | 6.94993 | 1656000 | P2RY8 |
| X | 48897478 | 6.94993 | 48897328 | TFE3 |
| 8 | 74503065 | 6.92246 | 74502809 | RP11-116B19.2 |
| 17 | 8129503 | 6.7339 | 8128798 | RP11-849F2.8 |
| 19 | 45981966 | 6.7339 | 45982086 | ERCC1 |
| MT | 9816 | 6.46829 | 9991 | MT-TG |
| 2 | 1.02E+08 | 6.1919 | 1.02E+08 | RNF149 |
| 6 | 26597056 | 5.78711 | 26597180 | ABT1 |
| 19 | 1383554 | 5.72421 | 1383526 | NDUFS7 |
| MT | 2643 | 5.67859 | 3230 | MT-TL1 |
| MT | 6287 | 5.50086 | 5904 | MT-CO1 |
| 5 | 95170432 | 5.4889 | 95170750 | AC008592.3 |
| 2 | 70476129 | 5.32642 | 70475793 | TIA1 |
| MT | 13045 | 4.8243 | 12337 | MT-ND5 |
| 2 | 60327418 | 4.54746 | 60327335 | RP11-444A22.1 |
| 21 | 37860550 | 4.54746 | 37859709 | PSMD4P1 |
| 7 | 99006474 | 4.54746 | 99006452 | PDAP1 |
| 15 | 65983337 | 4.1505 | 65982876 | DENND4A |
| 14 | 23504246 | 3.67256 | 23504354 | PSMB5 |
| MT | 10903 | 3.60378 | 10760 | MT-ND4 |
| 10 | 75634859 | 3.59911 | 75634342 | CAMK2G |
| 15 | 45492569 | 3.34481 | 45492830 | CTD-2651B20.6 |
| 18 | 51795615 | 3.34481 | 51795774 | POLI |
| 19 | 37619170 | 3.34481 | 37619069 | ZNF420 |
| 17 | 8090176 | 2.46085 | 8090493 | AC129492.1 |
| 17 | 56407997 | 2.38414 | 56408679 | MIR142 |
| 8 | 67025771 | 2.38414 | 67025522 | AC084082.3 |
| 8 | 9090340 | 2.3304 | 9090523 | RP11-10A14.8 |
| 15 | 41062072 | 1.74754 | 41062159 | C15orf62 |
| 7 | 1.39E+08 | 1.45893 | 1.39E+08 | C7orf55 |

CEBPE ChIP-Seq peaks called by MACS mapping within 1Kb of a transcription start site from Ensembl 90. Base positions from hg19.

**Supplementary Table 8: Position weight matrix for CEBPE.**

| Base  Position | A | C | G | T |
| --- | --- | --- | --- | --- |
| 1 | 0.425 | 0.127 | 0.394 | 0.054 |
| 2 | 0.001 | 0.001 | 0.001 | 0.997 |
| 3 | 0.001 | 0.001 | 0.001 | 0.997 |
| 4 | 0.090 | 0.001 | 0.751 | 0.158 |
| 5 | 0.072 | 0.822 | 0.001 | 0.105 |
| 6 | 0.267 | 0.054 | 0.625 | 0.054 |
| 7 | 0.162 | 0.639 | 0.001 | 0.198 |
| 8 | 0.986 | 0.012 | 0.001 | 0.001 |
| 9 | 0.997 | 0.001 | 0.001 | 0.001 |
| 10 | 0.001 | 0.443 | 0.217 | 0.339 |
| 11 | 0.356 | 0.268 | 0.072 | 0.304 |
| 12 | 0.159 | 0.307 | 0.302 | 0.231 |

Homer motif:

RTTGCGCAAYHN - *P*: 1 x10^-138^

*CEBPE* ChIP-Seq peaks called by MACS2 were analysed by HOMER v4.9.1 for motif enrichment, the most over represented DNA motif is shown. Each cell shows the probability of observing a specific base.

**Supplementary Table 9: CEBPE regulated genes**

| **Gene name** | **FDR *P*-Value** | | **Regulation by CEBPE** | **Fold change (log^2^)** | | **Distance to nearest**  **ChIP peak (Kb)** | **Location of nearest ChIP peak** | **HiC contact *P*-value**  **(Cell Line)** |
| --- | --- | --- | --- | --- | --- | --- | --- | --- |
|  | **DeSeq2** | **EdgeR** |  | **DeSeq2** | **EdgeR** |  |  |  |
| **CEBPE** | 3.34X10^-13^ | 1.40 X10^-14^ | na | -1.63094 | -1.71255 | n/a | n/a | n/a |
| **MPO** | 1.52X10^-10^ | 7.07X10^-11^ | +ve | -0.79375 | -0.90043 | 49.9 | chr17:56407997 | 3.9×10^-10^ (LCL) |
| **FAM69C** | 4.41X10^-09^ | 9.67X10^-08^ | +ve | -0.61221 | -0.70114 | 57.8 | chr18:72066694 | 7.4×10^-9^ (ESC) |
| **TRIM6-TRIM34** | 4.71X10^-09^ | 0.033783 | -ve | 21.68085 | 8.103069 | 4007.3 | chr11:1610669 | n/a |
| **PLEK** | 4.71X10^-09^ | 2.92X10^-10^ | +ve | -0.52247 | -0.60539 | 1861.1 | chr2:70476129 | n |
| **PLD1** | 3.98X10^-07^ | 8.18X10^-05^ | +ve | -0.7081 | -0.78477 | 12438.6 | chr3:183967364 | n/a |
| **PID1** | 4.12X10^-06^ | 4.22X10^-07^ | -ve | 0.711325 | 0.630334 | 3044.5 | chr2:233180511 | n/a |
| PCDH18 | 3.64X10^-05^ |  | -ve | 0.168791 |  | 1887.1 | chr4:140340770 | n |
| **LIG4** | 0.000136 | 0.00772 | -ve | 0.564279 | 0.516041 | 1840.4 | chr13:107026735 | n |
| **CPZ** | 0.000205 | 0.005736 | +ve | -0.40926 | -0.47393 | 433.6 | chr4:8160745 | n |
| **IRX3** | 0.000935 | 0.039769 | +ve | -0.47541 | -0.52545 | 3013.8 | chr16:57334463 | n/a |
| **DDIT4L** | 0.001443 | 3.85X10^-07^ | +ve | -0.52318 | -0.61207 | 1743.5 | chr4:99367970 | n |
| GNG7 | 0.003482 |  | -ve | 0.210746 |  | 1300.1 | chr19:1383554 | n |
| **FAM19A1** | 0.003701 | 0.011839 | -ve | 0.981966 | 0.839718 | 1121.5 | chr3:69417492 | n |
| FAM107B | 0.004086 |  | -ve | 0.29534 |  | 2042.9 | chr10:16859780 | n/a |
| SNX9 | 0.005526 |  | -ve | 0.395343 |  | 1216.2 | chr6:159460467 | n |
| **SLC2A5** | 0.005742 | 0.00772 | +ve | -0.42311 | -0.5041 | 2642.0 | chr1:11790564 | n/a |
| **KCNQ2** | 0.006352 | 0.001561 | -ve | 1.30335 | 1.239783 | 6065.4 | chr20:56000653 | n/a |
| ARHGEF6 | 0.006352 |  | +ve | -0.24511 |  | 70878.8 | chrX:64970732 | n/a |
| **ESAM** | 0.006352 | 0.003466 | -ve | 0.863719 | 0.83049 | 31032.3 | chr11:93595100 | n/a |
| **UGT3A2** | 0.006352 | 0.001561 | +ve | -0.29634 | -0.38037 | 12.1 | chr5:36054922 | too close |
| **KCNMB1** | 0.006847 | 0.024642 | -ve | 0.480428 | 0.408304 | 58.3 | chr5:169758363 | 7.6×10^-5^ (ESC) |
| **PRAME** | 0.00827 | 0.00079 | +ve | -0.75501 | -0.83778 | 293.0 | chr22:22603555 | n |
| **ICAM3** | 0.009712 | 0.012941 | -ve | 0.632008 | 0.568698 | 734.7 | chr19:11185230 | n |
| **FRG2B** | 0.013114 | 0.120114 | -ve | 0.523274 | 0.381051 | 367.5 | chr10:135072816 | n |
| **RASSF4** | 0.014254 | 0.055591 | +ve | -0.38107 | -0.4684 | 2924.2 | chr10:42530630 | n/a |
| MBP | 0.014254 |  | +ve | -0.20398 |  | 850.7 | chr18:73846776 | n |
| VOPP1 | 0.014254 |  | -ve | 0.228639 |  | 17849.7 | chr7:37734040 | n/a |
| SP4 | 0.014272 |  | -ve | 0.149719 |  | 10384.1 | chr7:31851863 | n/a |
| SLC30A4 | 0.017617 |  | +ve | -0.38407 |  | 321.9 | chr15:45492569 | n |
| TPM1 | 0.026621 |  | -ve | 0.348116 |  | 434.1 | chr15:63790137 | n |
| C4orf32 | 0.027812 |  | -ve | 0.303661 |  | 1.1 | chr4:113067619 | promoter |
| **FRG2C** | 0.029388 | 0.04452 | -ve | 0.781481 | 0.663449 | 6296.0 | chr3:69417492 | n/a |
| MDM2 | 0.036639 |  | -ve | 0.201757 |  | 1057.2 | chr12:70294638 | n |
| **MLC1** | 0.036639 | 0.000196 | +ve | -0.44395 | -0.51853 | 453.9 | chr22:50978250 | n |
| FAM72A | 0.041391 |  | -ve | 0.213056 |  | 1660.7 | chr1:204476244 | n |
| HS3ST1 | 0.044333 |  | -ve | 0.492067 |  | 51.2 | chr4:11482604 | 1.5×10^-4^ (LCL) |
| ANKRD36 | 0.046278 |  | +ve | -0.30907 |  | 4013.8 | chr2:101925426 | n/a |
| **KCNJ16** | 0.046278 | 0.070853 | -ve | 0.59322 | 0.492369 | 1706.8 | chr17:66342817 | n |
| IL7R | 0.046278 |  | +ve | -0.19333 |  | 179.4 | chr5:36054922 | n |
| EFNB1 | 0.046278 |  | +ve | -0.2666 |  | 3078.1 | chrX:64970732 | n/a |
| TNFRSF1B | 0.046975 |  | +ve | -0.52082 |  | 461.3 | chr1:11790564 | n |
| VEZF1 | 0.046975 |  | +ve | -0.17035 |  | 342.4 | chr17:56407997 | 1.5×10^-13^ (LCL) |
| NUCB2 | 0.053829 |  | +ve | -0.15865 |  | 2687.7 | chr11:14542009 | n/a |
| **TUBA4A** | 0.054117 | 0.066839 | -ve | 0.433179 | 0.352245 | 3080.9 | chr2:223223799 | n/a |
| BCL2 | 0.058521 |  | +ve | -0.44932 |  | 61.6 | chr18:60767513 | 1.9×10^-107^ (ESC) |
| **QRSL1** | 0.059195 | 0.061149 | -ve | 0.409968 | 0.334686 | 4819.3 | chr6:111896831 | n/a |
| **HIST1H2BK** | 0.063954 | 0.073238 | -ve | 0.491465 | 0.392883 | 517.5 | chr6:26597061 | 1.3×10^-52^ (LCL) |
| CDH12 | 0.065231 |  | -ve | 0.526414 |  | 8.5 | chr5:22862278 | too close |
| UCK2 | 0.072861 |  | -ve | 0.179466 |  | 4286.7 | chr1:161510027 | n/a |
| **PDE1A** | 0.075045 | 0.117936 | -ve | 1.303618 | 1.218924 | 4978.4 | chr2:178128223 | n/a |
| MSANTD3-  TMEFF1 |  | 1.89X10^-06^ | -ve |  | 7.823011 | 4426.5 | chr9:107631070 | n/a |
| PEAR1 |  | 0.00772 | +ve |  | -1.50876 | 1256.0 | chr1:155607443 | n |
| ABCA4 |  | 0.00772 | +ve |  | -0.5996 | 4314.7 | chr1:90158578 | n/a |
| DIRC3 |  | 0.012941 | +ve |  | -1.17514 | 1436.3 | chr2:216862590 | n |
| IL1B |  | 0.012941 | +ve |  | -0.48747 | 3736.0 | chr2:109855160 | n/a |
| CHRD |  | 0.047118 | +ve |  | -0.93088 | 130.6 | chr3:183967364 | n |
| PAX7 |  | 0.054725 | +ve |  | -1.21703 | 4899.9 | chr1:23857894 | n/a |
| SERPING1 |  | 0.061432 | +ve |  | -0.32136 | 1939.8 | chr11:59318458 | n |
| TOX |  | 0.070853 | -ve |  | 1.58381 | 6994.0 | chr8:67025771 | n/a |
| REG1A |  | 0.070853 | -ve |  | 1.490084 | 8871.4 | chr2:70476129 | n/a |
| MKX |  | 0.098183 | -ve |  | 0.970422 | 6018.8 | chr10:22013722 | n/a |
| AGR2 |  | 0.098183 | -ve |  | 1.015929 | 11403.9 | chr7:5436920 | n/a |
| LHFP |  | 0.10145 | +ve |  | -0.41908 | 151.0 | chr13:40026312 | 3.3×10^-34^ (ESC) |
| MSH5-SAPCD1 |  | 0.104742 | +ve |  | -0.8626 | 426.4 | chr6:31300805 | 9.3×10^-3^ (LCL) |
| SLC9A9 |  | 0.111622 | -ve |  | 0.325787 | 11261.5 | chr3:154828861 | n/a |
| SMN2 |  | 0.111684 | +ve |  | -0.6174 | 7519.7 | chr5:76886229 | n/a |
| RP11-552F3.12 |  | 0.117302 | +ve |  | -1.86072 | 878.5 | chr17:73031281 | n |
| PPAN-P2RY11 |  | 0.118616 | +ve |  | -6.51753 | 968.2 | chr19:11185230 | n |
| NDFIP2 |  | 0.118676 | +ve |  | -0.37818 | 18032.0 | chr13:98125164 | n/a |
| FAM57B |  | 0.134301 | +ve |  | -0.56754 | 7732.3 | chr16:22308540 | n/a |
| RANBP3L |  | 0.134301 | +ve |  | -0.97114 | 102.1 | chr5:36404304 | 3.2×10^-19^ (ESC) |
| SLC16A11 |  | 0.144043 | -ve |  | 1.670349 | 1012.5 | chr17:7959699 | n |
| CAPG |  | 0.155394 | +ve |  | -0.27369 | 15149.3 | chr2:70476129 | n/a |

The top 73 *CEBPE* regulated genes determined by EdgeR and DeSeq2, ordered by Benjamini-Hochberg corrected *P*-Values. Table created by merging the top 50 differentially expressed genes in called by either program, 27 duplicate genes were merged and shown in bold and underlined. The ‘Regulation by CEBPE’ column shows whether *CEBPE* depletion was associated with decreased expression (+ve regulation) or increased (-ve regulation). The distance from each gene transcription start site and the nearest called CEBPE ChIP-seq peak is listed in Kb and with genetic coordinates from hg19. When a differentially expressed gene occurred within 2Mb of a *CEBPE* ChIP peak the presence of a DNA looping interaction with the gene TSS was assessed using HiC data from the HUGIn browser (http://yunliweb.its.unc.edu/HUGIn/). Lymphoblastoid cell line GM12878 (LCL) and embryonic stems cells (ESC) were investigated and the *P*-value of the most significant contact in either is listed, the absence of a significant contact denoted by ‘no’. Instances where the distance between the gene TSS and the nearest ChIP peak was less than the resolution of HiC are denoted by ‘too close’. Genes further than 2Mb from the nearest ChIP peak were not investigated and are denoted with ‘n/a’. Promoter ChIP-peaks are denoted by ‘promoter’.

**Supplementary Table 10 : MotifbreakR output for rs2239635**

| Rank | Gene Symbol | ProviderName | Pct(max) – pct(min) |
| --- | --- | --- | --- |
| 1 | TFAP2A | TFAP2_known2 | 0.288696 |
| 2 | TFAP2A | TFAP2_known8 | 0.288696 |
| 3 | ZBTB7A | ZBTB7A_known1 | 0.280146 |
| 4 | TFAP2C | TFAP2_known3 | 0.269389 |
| 5 | TFAP2A | TFAP2_known9 | 0.232318 |
| 6 | TEAD2 | TEAD2_known_ref1 | 0.232081 |
| 7 | RAR | RAR_known_ref1 | 0.225663 |
| 8 | REST | REST_disc8 | 0.223869 |
| 9 | TFAP2C | TFAP2_known10 | 0.218162 |
| 10 | ZNF711 | ZNF711(Zf)/SHSY5Y-ZNF711-ChIP-Seq(GSE20673)/Homer | 0.202075 |
| 11 | TFAP2C | AP-2gamma(AP2)/MCF7-TFAP2C-ChIP-Seq(GSE21234)/Homer | 0.199654 |
| 12 | NA | AP2 | 0.193808 |
| 13 | PPARG | PPARG_si | 0.192301 |
| 14 | TFAP2E | TFAP2E_known_ref1 | 0.189729 |
| 15 | ESR2 | ESR2_si | 0.185145 |
| 16 | PLAGL1 | PLAGL1_known_ref1 | 0.183128 |
| 17 | ZFX | ZFX(Zf)/mES-Zfx-ChIP-Seq(GSE11431)/Homer | 0.181465 |
| 18 | PLAG1 | PLAG1_f1 | 0.178707 |
| 19 | EBF1 | COE1_f2 | 0.178385 |
| 20 | ZFX | ZFX_f1 | 0.176156 |
| 21 | TFAP2A | TFAP2_known6 | 0.175142 |
| 22 | ZFX | ZFX_known_ref1 | 0.171627 |
| 23 | FLI1 | FLI1_f1 | 0.167695 |
| 24 | NA | EBF1 | 0.165919 |
| 25 | NFIA+NFIB+NFIC+NFIX | NFIA+NFIB+NFIC+NFIX_si | 0.164496 |
| 26 | TFAP2 | TFAP2_known5 | 0.161365 |
| 27 | PPARA | PPARA_f1 | 0.16102 |
| 28 | NA | EBF1(EBF)/Near-E2A-ChIP-Seq(GSE21512)/Homer | 0.160644 |
| 29 | GLI2 | GLI2_known_ref1 | 0.158073 |
| 30 | EBF1 | EBF1_disc1 | 0.15505 |
| 31 | EBF1 | EBF(EBF)/proBcell-EBF-ChIP-Seq(GSE21978)/Homer | 0.152619 |
| 32 | SP1 | SP1_known6 | 0.152438 |
| 33 | EBF1 | EBF1_known2 | 0.149405 |
| 34 | SP1 | SP1_known5 | 0.149151 |
| 35 | NFIC | NFIC_known_ref2 | 0.149042 |
| 36 | ZNF423 | ZNF423_known_ref2 | 0.148126 |
| 37 | TFAP2B | TFAP2B_known_ref1 | 0.14325 |
| 38 | EBF1 | EBF1_known1 | 0.139081 |
| 39 | SPZ1 | SPZ1_known_ref1 | 0.136507 |
| 40 | EBF1 | EBF1_known3 | 0.130992 |
| 41 | NFIC | NFIC_known_ref4 | 0.127357 |
| 42 | EBF1 | EBF1_known4 | 0.126319 |
| 43 | TFAP2C | TFAP2_known14 | 0.122433 |
| 44 | RXRA | RXRA_f1 | 0.121536 |
| 45 | TFAP2B | TFAP2B_known_ref2 | 0.119721 |
| 46 | ITGB2 | ITGB2_known_ref1 | 0.115694 |
| 47 | NR4A2 | NR4A_known1 | 0.110114 |
| 48 | REST | REST_disc5 | 0.105807 |
| 49 | TFAP2B | AP2B_f1 | 0.102786 |
| 50 | GLI | GLI_known_ref1 | 0.102324 |
| 51 | TFAP2A | TFAP2_known11 | 0.101298 |
| 52 | TFAP2A | TFAP2_known20 | 0.098265 |
| 53 | GLI1 | GLI1_f1 | 0.095736 |
| 54 | INSM1 | INSM1_known_ref1 | 0.089375 |
| 55 | NR2F2 | COT2_f2 | 0.087617 |
| 56 | TFAP2C | TFAP2_known17 | 0.080253 |
| 57 | REST | REST_disc4 | 0.069875 |
| 58 | NRF1 | NRF1_disc3 | 0.069466 |
| 59 | ZIC1 | ZIC1_f1 | 0.067401 |
| 60 | TFAP2 | TFAP2_known1 | 0.0553 |
| 61 | HNF4A | HNF4_known10 | 0.054127 |
| 62 | **IKZF1** | **IKZF1_known_ref2** | **0.052582** |
| 63 | HNF4A | HNF4_known11 | 0.050652 |
| 64 | HES1 | HES1_f1 | 0.048964 |
| 65 | RAD21 | RAD21_disc10 | 0.047833 |
| 66 | ZBTB7A | ZBT7A_f1 | 0.047723 |
| 67 | ZIC3 | ZIC3_f1 | 0.037875 |
| 68 | MYC::MAX | MYC_known8 | 0.026386 |

*In silico* prediction of transcription factor motif disruption associated with rs2239635 genotype performed using MotifbreakR (3). Transcription factors are listed according to the difference in pct(max) – pct (min). Pct values estimate avidity of TF binding at each allele. IKZF1 (shown in bold) ranks 62 of 68 altered motifs.

**
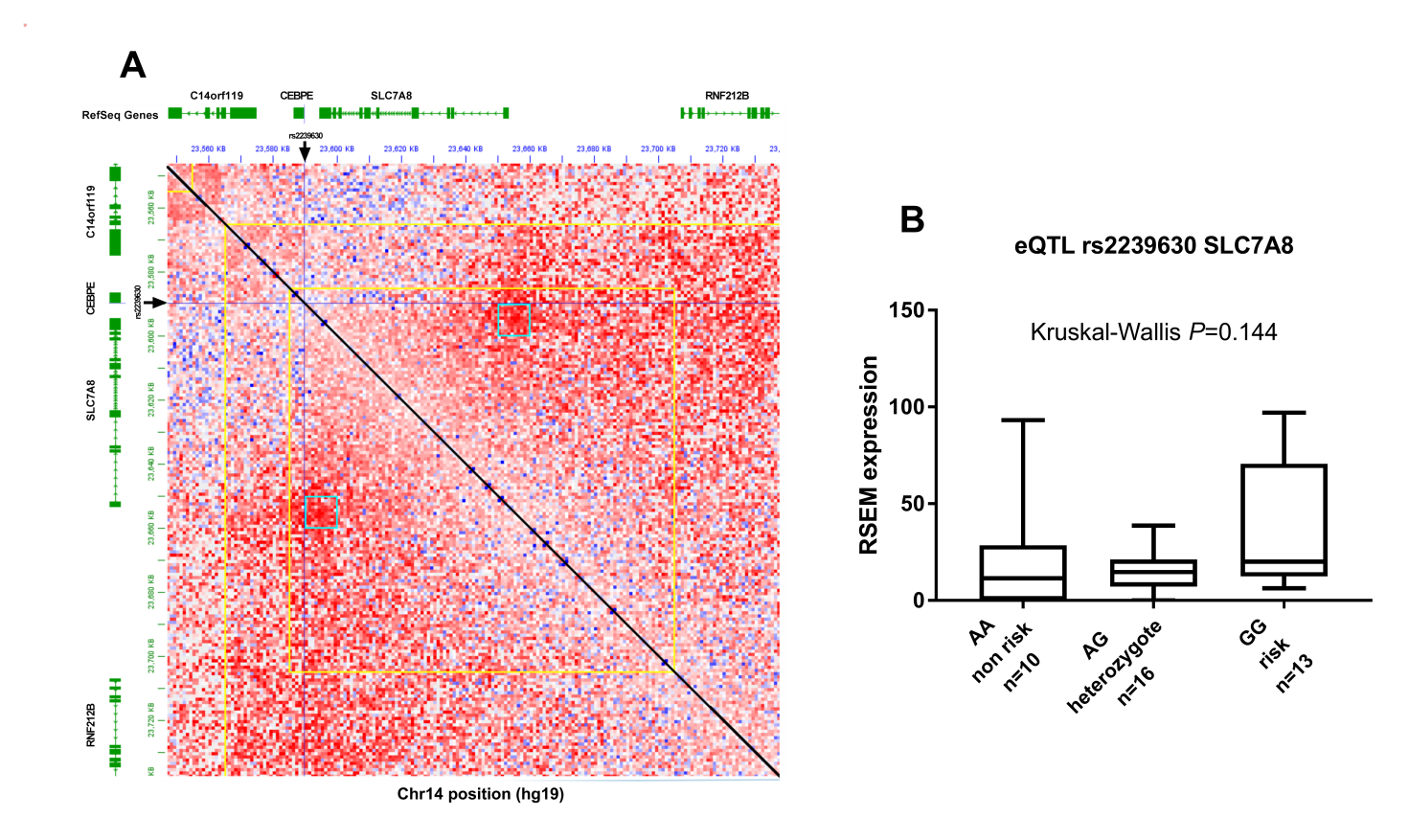
**

**Supplementary Figure 1:** **Risk allele of rs2239630 is not associated with *SLC7A8* expression** (**A**) HiC heat map, at 5kb resolution, based on data from Rao et al(4). Enriched interaction frequencies are shown in red and depleted interactions in blue. Data from combined MboI experiments in GM12878, shown using Knight-Ruiz normalised observed/expected interaction frequencies. Topologically interacting domains and contacts, as called in Rao et al, are shown in yellow and light blue boxes respectively. The location of the lead risk SNP rs2239630 is highlighted by arrows and vertical blue lines. Image downloaded (https://www.aidenlab.org/juicebox/). (**B**) Box and whiskers plot (min, max) of expression quantitative trait loci (eQTL) analysis between rs2239630 and *SLC7A8* performed in ALL disomic for chromosome 14. *P*-value calculated using Kruskal-Wallis test in R. Figure generated in GraphPad v7.

**
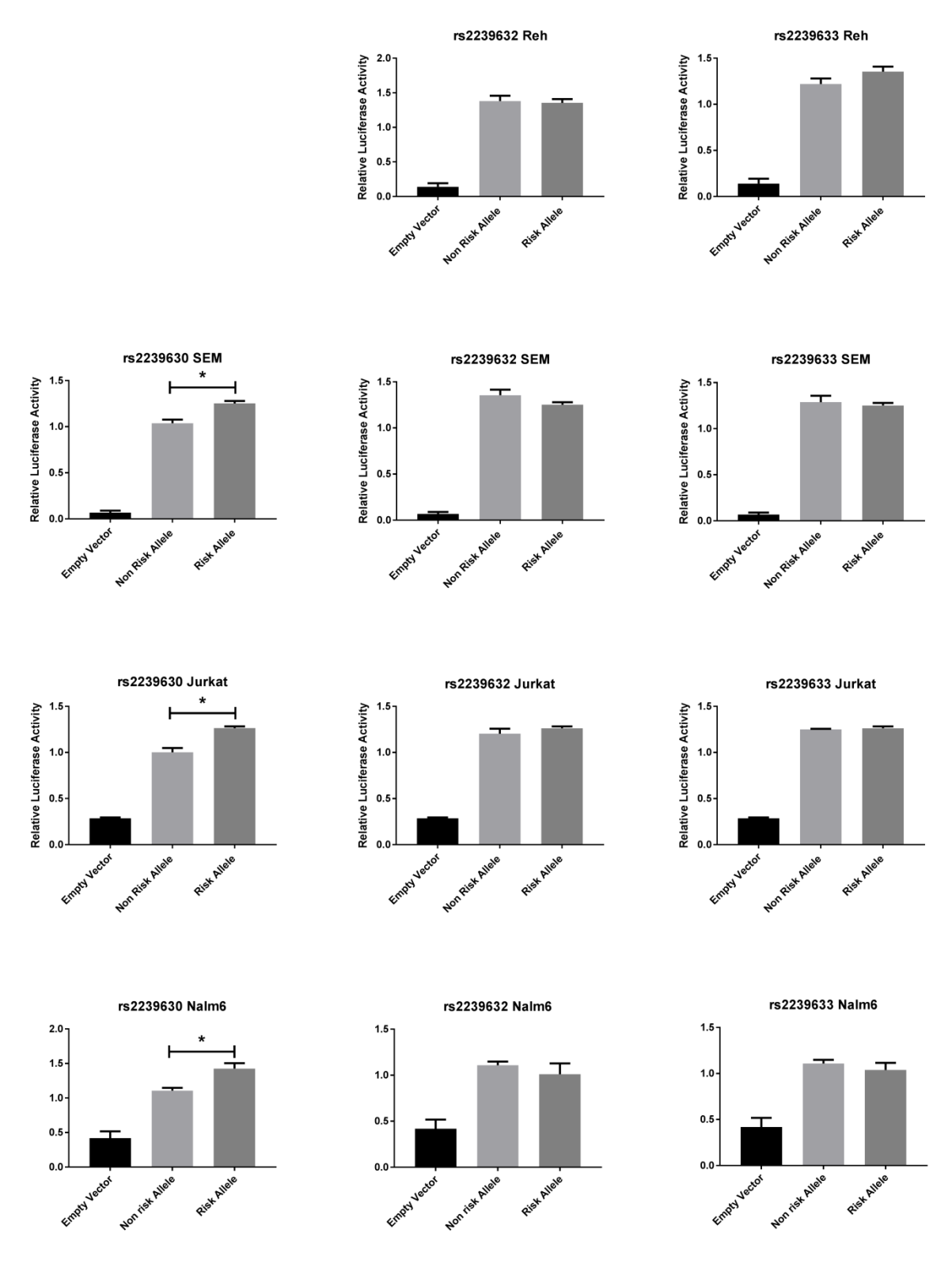
**

**Supplementary Figure 2: rs2239630 but not rs2239632 rs2239633 influences *CEBPE* promoter activity.** (**A**) *CEBPE* promoter reporter assay. The promoter of *CEBPE* with risk SNP allelic variants was cloned into pGL3 reporter vectors and transfected in REH, SEM and Jurkat cells (n=3). The ratio of luminescence from the experimental pGL3 constructs was normalised to the Renilla internal control, pRL-SV40. Significance testing preformed using Student’s T-test * denotes *P*<0.05>0.01. Figures generate in Graph Pad v7.

**
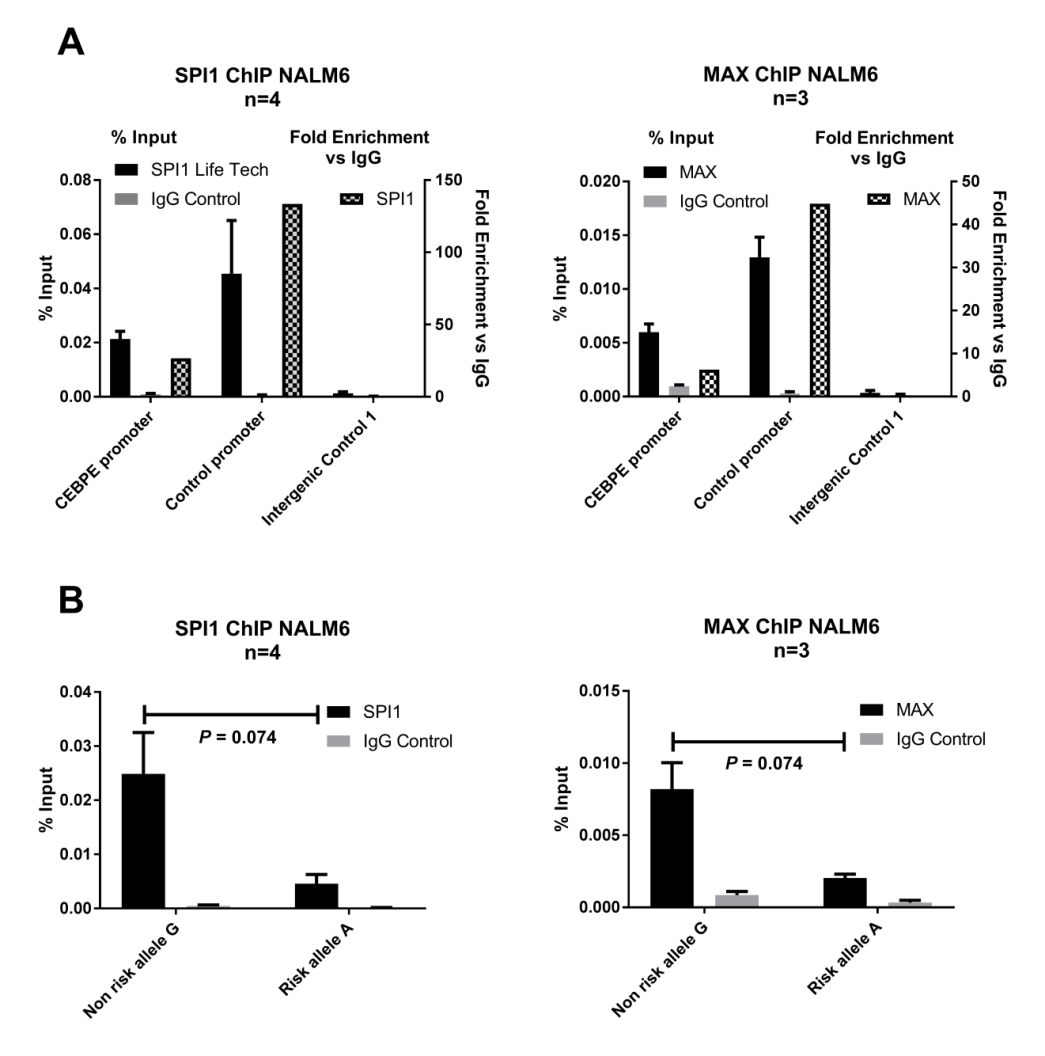
**

**Supplementary Figure 3: MAX and SPI1 bind rs2239630. ChIP q-PCR in NALM6.** (**A**) ChIP’d DNA was amplified with primers for either; the promoter of *CEBPE*, an antibody specific promoter positive control or an intergenic negative control. Left *y*-axis shows signal as percent of input DNA for specific antibody (solid black bar) or IgG isotype control (solid grey bar). Right *y-*axis shows fold enrichment for specific antibody over IgG (checked box). Figures produced in Graph Pad v7. (**B**) Allele specific ChIP q-PCR for MAX and SPI1 in NAML6. ChIP’d DNA was amplified with primers specific for either allele of rs2239630, signal shown as percent of input DNA.


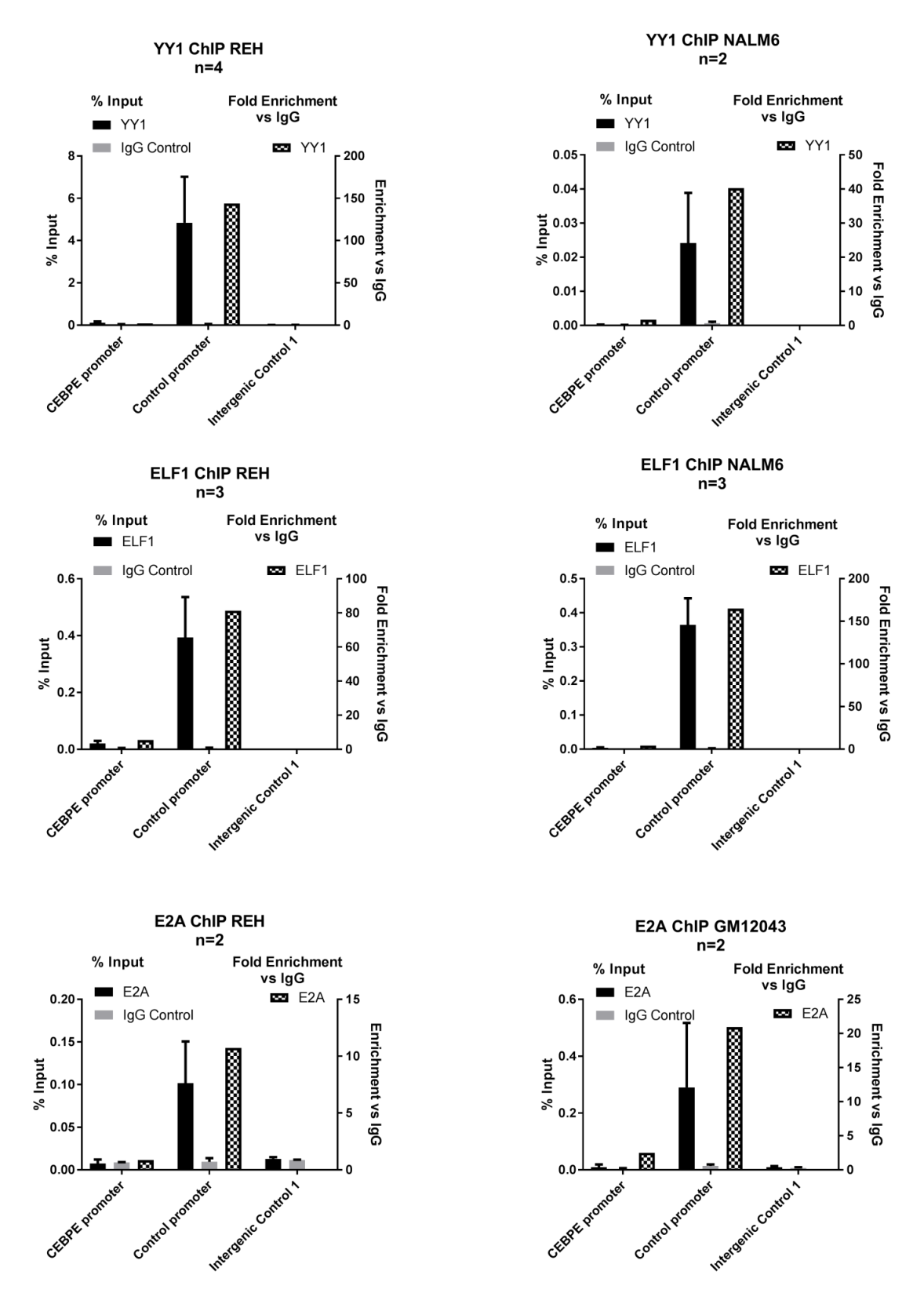


**Supplementary Figure 4: YY1, ELF1 and E2A do not bind rs2239630. ChIP q-PCR in REH, NALM6 and GM12043.** ChIP’d DNA was amplified with primers for either; the promoter of *CEBPE*, an antibody specific promoter positive control or an intergenic negative control. Left *y*-axis shows signal as percent of input DNA for specific antibody (solid black bar) or IgG isotype control (solid grey bar). Right *y*-axis shows fold enrichment for specific antibody over IgG (checked box). Figures produced in Graph Pad v7.


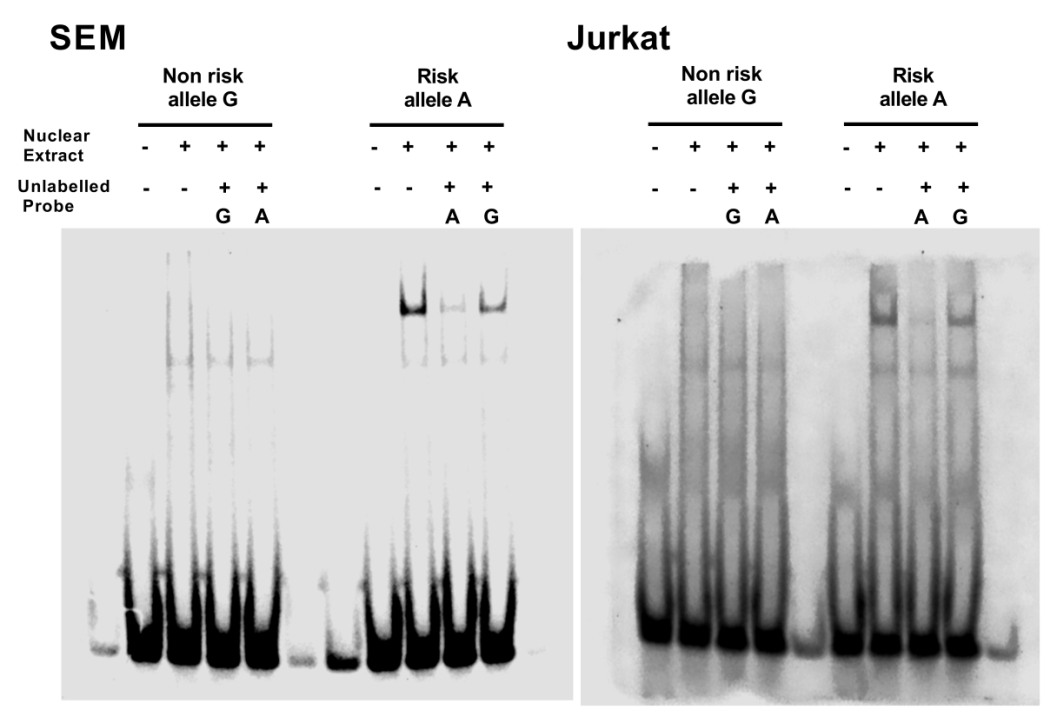


**Supplementary Figure 5: Alleles of rs2239630 differentially bind protein.** Electrophoretic mobility shift assay (EMSA) using probes flanking rs2239630, showing differential allelic binding of nuclear protein extract from SEM and Jurkat cells. Competition assays performed by the addition of a 100-fold excess of unlabelled probe.

**Supplementary Figure 6: *CEBPE* knock-down has no effect on the cell cycle, background or induced apoptosis.** (**A**) 168 hours after induction with 1µM doxycycline cells were fixed, labelled with propidium iodide and analysed by flow cytometry. The columns show the stacked %age of cells in each stage of the cell cycle. Sub G0 - apoptotic population, G1 – growth 1 phase, S – synthesis phase, G2 – growth 2 phase. (**B,C,D**) After induction with 1µM doxycycline cells were labelled with DAPI and an annexin V antibody and analysed by flow cytometry. Cells were divided into 4 populations based on the annexin-V and DAPI positivity/negativity.


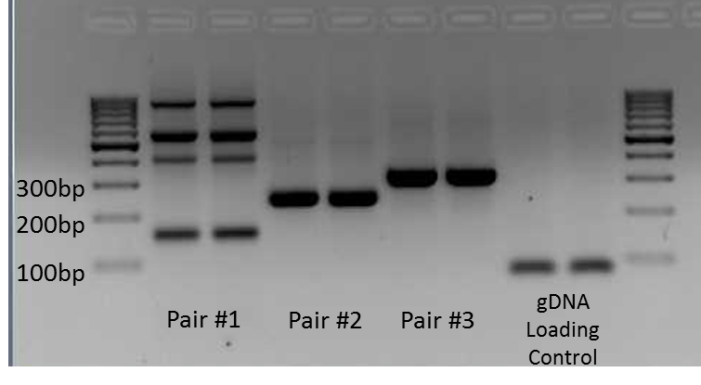


**Supplementary Figure 7 :** PCR gel image using primers spaing CEBPE and IGH breakpoints in the Singapore cohort t(14:14) ALL case, amplified from genomic DNA. PCR product size indicates absence of addiitional intronic sequences between breakpoints. Primers; Pair #1: IGHJ4 fwd + CEBPE exon 2, Pair #2: IGHJ4 upstream + CEBPE exon 2, Pair #3: IGHJ4 upstream + CEBPE exon 2 mk2. Primer sequences detailed in **Supplementary Table 1**.


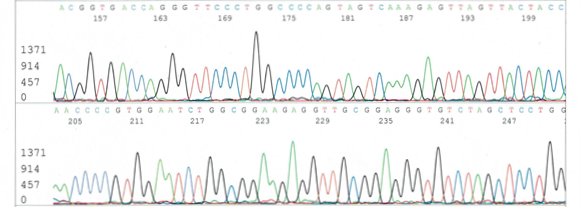


**CEBPE E2**

**IgHJ4 JjJ4**

**Supplementary Figure 8:** Sanger sequencing electropherogram from Singapore cohort t(14:14) ALL case of genomic PCR product ‘Pair #3’ spanning CEBPE/IGH breakpoints using primer ‘IGH4 upstream mk2’ detailed in **Supplementary Table 1**.

**Supplementary Figure 9: Regional association plot for 14q11.2.** (**A**) SNPs plotted by GWAS *P*-values (−log_10_, *y*-axis) in red circles (upper pane) or eQTL *P*-values (−log_10_, *y*-axis) in green (MuTHER *P*-value) or blue circles (Blood FDR corrected *P*-value) (lower pane) and by location (*x*-axis, GRCh37/hg19). Recombination rate (cM/Mb) - light blue line (right *y*-axis). Linkage disequilibrium with lead SNP, (r^2^ = 0, white, r^2^ = 1.0, dark red). (**B**) ENCODE ChIP-seq peaks, ChromHMM chromatin state segmentation in GM12878 LCL and Roadmap B-cell E031 and DNAse hypersensitivity (darkness indicates intensity of peak) from GM12878 and Blueprint ALL blast sample pz294 in relation to rs2239630 and rs2239635 (vertical dashed lines).

**Supplementary acknowledgments**

The 14q11.2 risk locus was originally identified by Fay J. Hosking, Yussanne Ma and Amy L. Sherborne; Institute of Cancer Research, United Kingdom. Bettina Fiege, Rajiv Kumar, Miguel Inacio da Silva Filho, Hauke Thomsen and Kari Hemminki; German Cancer Research Centre, Heidelberg, Germany. Julie A. Irving and James M. Allan; Northern Institute for Cancer Research, Newcastle University, United Kingdom. Per Hoffmann, Markus M. Nöthen and Thomas W. Mühleisen; Institute of Human Genetics, University of Bonn, Germany. Lewin Eisele; Institute for Medical Informatics, Biometry and Epidemiology, University Hospital Essen, University of Duisburg–Essen, Essen, Germany. Martin Zimmermann and Martin Stanulla; Department of Pediatric Hematology and Oncology, Hannover Medical School, Hannover, Germany. Martin Schrappe; General Pediatrics, University Hospital Schleswig-Holstein, Kiel, Germany.

**References**

1. Ward LD, Kellis M. HaploReg: A resource for exploring chromatin states, conservation, and regulatory motif alterations within sets of genetically linked variants. Nucleic Acids Res. Oxford University Press; 2012 Jan;40(D1):D930-4.

2. Boyle AP, Hong EL, Hariharan M, Cheng Y, Schaub MA, Kasowski M, et al. Annotation of functional variation in personal genomes using RegulomeDB. Genome Res. 2012;22(9):1790–7.

3. Coetzee SG, Coetzee GA, Hazelett DJ. *motifbreakR* : an R/Bioconductor package for predicting variant effects at transcription factor binding sites: Fig. 1. Bioinformatics. 2015 Aug 12;31(23):btv470.

4. Rao SSP, Huntley MH, Durand NC, Stamenova EK, Bochkov ID, Robinson JT, et al. A 3D map of the human genome at kilobase resolution reveals principles of chromatin looping. Cell. Cell Press; 2014;159(7):1665–80.
